# The Dark Side of “Smart Drugs”: Cognitive Enhancement vs. Clinical Concerns

**DOI:** 10.3390/toxics13040247

**Published:** 2025-03-26

**Authors:** Mariarosaria Ingegneri, Erika Smeriglio, Younes Zebbiche, Laura Cornara, Letterio Visalli, Antonella Smeriglio, Domenico Trombetta

**Affiliations:** 1 Department of Chemical, Biological, Pharmaceutical and Environmental Sciences (CHIBIOFARAM), University of Messina, Viale Ferdinando Stagno d’Alcontres 31, 98166 Messina, Italy; mariarosaria.ingegneri@unime.it (M.I.); domenico.trombetta@unime.it (D.T.); 2 Department of Cognitive, Psychological and Pedagogical Sciences, and Cultural Studies (COSPECS), University of Messina, Via Concezione 6/8, 98121 Messina, Italy; erika.smeriglio@studenti.unime.it; 3 Faculty of Pharmacy, University of Algiers, Algiers 16002, Algeria; y.zebbiche@univ-alger.dz; 4 National Center of Toxicology Algiers, Algiers 16062, Algeria; 5 Department of Earth, Environment and Life Sciences (DISTAV), University of Genova, Corso Europa 26, 16132 Genova, Italy; laura.cornara@unige.it; 6 Health Service Department, Ministry of Interior, 00185 Rome, Italy; visallimedleg@gmail.com

**Keywords:** legal highs, micromorphology, DNA barcoding, chemical analysis, cognitive enhancers, mood enhancers, neuropharmacological effects, psychological implications, cognitive decline, adverse effects

## Abstract

The European Union Drugs Agency has emphasized the increasing difficulty in monitoring the drug market due to the emergence of new psychoactive substances, often marketed as legal highs. The proliferation of fake pharmacies, drugstores, and e-commerce platforms has made access to illicit substances alarmingly rapid and inexpensive. These substances are readily available without medical prescriptions, lacking proper risk assessments or monitoring of potential adverse effects, raising significant public health concerns. Today, the relentless pursuit of validation and success—often, at any cost—has led to an exponential rise in the use of cognitive and mood enhancers. Such substances are frequently consumed to manage demands related to work, diet, sexuality, sleep, achievement, and interpersonal relationships. Consequently, investigating these phenomena is critically important for institutions, as they represent a serious threat to individual development and health. Developing effective preventive and protective systems is essential. This review provides an overview of currently available smart drugs, discussing their desired and adverse neuropharmacological effects, psychological implications, and cognitive decline resulting from their excessive and unregulated use. This review concludes that a multidisciplinary approach combining molecular identification, micro-morphological analysis, and chemical characterization is crucial for the accurate detection, monitoring, and risk mitigation of new psychoactive substances.

## 1. Introduction

Since the European Monitoring Centre for Drugs and Drug Addiction (EMCDDA) was founded in 1993, the scale and nature of drug addiction have changed significantly. To address new challenges, mostly related to the phenomenon of poly-drug use of classic abuse substances, including alcohol and new psychoactive substances, the mandate of the agency was revised, and on 2 July 2024, the EMCDDA was converted into the European Union Drugs Agency (EUDA) [1]. Major concerns in this regard include *Cannabis* products adulterated with synthetic cannabinoids, products sold as 3,4-methylenedioxymethamphetamine (MDMA or ecstasy) but sometimes containing synthetic cathinones as adulterants, and the emergence of highly potent synthetic opioids mis-mixed or sold as other substances. It is also important to note that the combined use of alcohol with illicit drugs can also increase health risks, for example, when alcohol is taken in combination with cocaine, opioids, or new or “street” benzodiazepines [2].

In this context, it is important to clarify the terminology used in this review. The terms “cognitive enhancers” and “mood enhancers” refer broadly to substances that enhance cognitive functions (e.g., attention, memory, and decision-making abilities) or positively influence mood (reducing anxiety, stress, etc.). However, the term “smart drugs” specifically denotes psychoactive substances—natural or synthetic—that are often used for cognitive or mood enhancement purposes but are also characterized by significant adverse effects, risks of abuse, dependency, or potential neurotoxicity, typically used without appropriate medical supervision. Therefore, not all cognitive and mood enhancers can be categorized as smart drugs, and this distinction is essential for accurately understanding the risks and benefits associated with these substances.

For instance, synthetic cannabinoids, often marketed under various street names (such as Spice or K2), have rapidly become popular in many regions, particularly among adolescents and young adults. Their widespread availability and misleading labels have contributed to their use, posing considerable health risks, including severe psychosis, anxiety, tachycardia, seizures, and even fatalities [3,4]. Similarly, synthetic cathinones, commonly known as “bath salts”, have gained notoriety globally due to their stimulant and hallucinogenic properties. Their easy accessibility, low cost, and potent effects have led to numerous adverse outcomes in large populations, such as acute intoxications, cardiovascular toxicity, and severe psychiatric disturbances [5,6]. Conversely, some plant-derived compounds, like *Mitragyna speciosa* Korth. (kratom), exhibit both potential therapeutic benefits (e.g., analgesic properties and management of opioid withdrawal symptoms) and substantial health risks associated with misuse and dependency [7,8]. These examples underscore the complexity of assessing the impact of various cognitive and mood enhancers at the population level, necessitating ongoing research and monitoring efforts to provide clear evidence-based recommendations for public health [9].

The EUDA aims to support the European Union and its Member States, improving and extending the monitoring of drug use and related concerns, investing in the development of new skills that can enable a faster identification of new threats, and consequently, a faster response, preserving health and improving safety [1]. In Italy, the Istituto Superiore di Sanità (ISS), the national reference in the European Early Warning System (SNAP) network on new psychoactive substances, studies and disseminates information on new trends in the consumption of classic drugs and new psychotropic substances, as well as the effectiveness of preventive, therapeutic, and rehabilitative interventions in the field of drug addiction [10]. In recent years, as highlighted by the Italian Medicines Agency (AIFA), the Internet and illegal purchases have certainly cannibalized the market for narcotic substances but not only that. In our country, approximately 500 web portals have been identified, not necessarily based in Italy, that sell this type of substance, and 64% of these sites have been permanently closed [11]. The network of fake pharmacies, drugstores, and e-commerce sites makes obtaining illicit substances dramatically quick and cheap. No medical prescriptions are needed, and there is no monitoring of risks, and in general of the consequences of their administration [11]. According to a very recent study [12], many new substances are marketed as legal highs, even when the indications include the wording “not intended for human consumption”; in some cases, in fact, to avoid controls, new drugs are produced in clandestine laboratories in Europe and around the world and sold directly on the market with labels containing misleading information, for example, “chemical substances intended for research” or “fertilizers”.

In its latest 2024 report, the EUDA states that the analysis of this market highlights how it is becoming increasingly difficult to examine this segment due to the continuous emergence of new psychoactive substances designed to mimic the effects typically caused by drugs already included in the prohibited schedules [2]. The analysis of indicators related to the supply of commonly used illicit drugs in the European Union suggests that availability remains high for almost all types of substances. Furthermore, available information suggests that the market is now characterized by widespread availability of a wider range of drugs than in the past, with substances often available at high potency or purity or in new forms, mixtures, or combinations. These include new substances, for which both consumer and scientific knowledge about the health risks may be limited. There is also a growing diversity in the forms in which these substances may be available on the market and, in some cases, also in the routes of administration by which they can be consumed, with edibles and various forms of vaping technologies. These developments raise concerns that the risks associated with some substances, including overdose, may be increasing. Specifically, cognitive enhancers, substances used to increase certain mental functions such as attention, memory, concentration, motivation, planning, self-esteem, and decision-making ability, are among the most worrying [13]. In fact, while they increase cognitive abilities and reduce feelings of fatigue, sleep, and hunger, on the other hand, they cause harm on a par with real drugs, although smart drugs are apparently not always illegal and therefore law prosecutable [13].

In a society that is always running faster, the ambition to surpass and improve performance knows no limits, even when there is a clear danger to health. Pressure, expectations, and anxiety are therefore faced without considering the devastating effects that the intake of certain products can cause. People are never thin, smart, reactive, cheerful, or flexible enough, but rather than stopping to reflect on and solve the problems, they take refuge in solutions that are as quick as they are often harmful. Viral advertising also pushes us to constantly rethink our idea of psychophysical well-being, pushing beyond the limits of our capabilities. The pyramid theory of primary needs formulated by the American psychologist Abraham Maslow (1962), which placed physiological needs at the base (food, sleep, etc.), followed by safety needs (protection and stability); psychological needs (belonging, esteem, and competence); and, finally, the self-realization need, considered not as an impossible or morally questionable goal to achieve but as the ultimate and complete achievement (top of the pyramid), seems today more than ever outdated [14]. Today, primary needs have been replaced by the need to assert and legitimize us by any means, at any cost, and in the shortest time possible. This inevitably leads to an exponential increase in the use of narcotic substances to deal with everything, from waking up to work, through food, sex, sleep, success, family, and interpersonal relationships.

The youth universe, in this sense, is constantly evolving, and as such, the manifestation of the problems that young people experience changes suddenly and unexpectedly. For institutions, it is of fundamental importance to study those phenomena that seriously jeopardize their development and growth and can compromise their health. In our society, in which it is now evident that adolescents represent one of the most at-risk groups, even more so if they belong to more disadvantaged social and territorial realities, it is essential to prepare an effective protection system [15].

From this perspective, it becomes a priority, on the one hand, to have an in-depth knowledge of the risks related to the consumption of more or less legal substances that our young people easily find in the environments they frequent and, on the other, to identify interventions to combat youth distress that prevent the spread of phenomena such as the consumption of narcotic substances and alcohol abuse. For this reason, it is important to promote the study and research on smart drugs (a new psychoactive substances used non-medically for cognitive or mood enhancement purposes). The aim is not only to provide adults of reference, such as educators and family members, with the tools needed to be aware of and informed about the risks that our young people encounter daily but, also and above all, to stimulate the young people themselves to play an active role in society. The hidden potential to improve life lies in helping young people to rediscover the desire to live, to recognize their own needs and desires, principles rooted in human existence [16]. The dual objective that we intend to achieve is, therefore, to inform young people about the risks deriving from the use of these substances, which often have attractive forms and effects but also to work on their personal growth and the promotion of healthy lifestyles. Knowledge is certainly important to promote awareness of actions and behaviors, but equally vital is a preventive intervention that intercepts problems when they arise, stopping, in time, harmful and damaging behaviors for oneself and others [15].

## 2. Search Strategy

The approach for this review was primarily based on a combination of data from national and international reference centers for drugs and drug addiction, including the EMCDDA, EUDA, SNAP (ISS), and AIFA, along with all data available from the published literature. To this end, several databases were consulted, including PubMed, Scopus, EMBASE, and MEDLINE, using the following keywords: smart drugs; herbal smart drugs; legal highs; cognitive enhancers; mood enhancers; smart drugs and neuropharmacological effects; smart drugs and adverse effects; smart drugs analysis; clinical impact and smart drug abuse; smart drugs and ethical costs.

## 3. Old and New Smart Drugs

### 3.1. Plant-Derived Smart Drugs

New psychoactive drugs represent an emerging class of substances whose number, variety, and availability have been steadily increasing in recent years. These substances, commonly referred to as “legal highs”, smart drugs, “research chemicals”, or “bath salts”, can mimic the psychoactive effects of illicit drugs; yet, in most cases, they remain unregulated [17,18]. Smart drugs can be either natural or synthetic [19].

Among drug users, a particularly widespread trend is the consumption of “herbal highs”, which are plant-derived products containing psychoactive substances [20]. New psychoactive substances are primarily composed of plant alkaloids, which induce novel sensations and altered mental states in those who consume them [19]. The plant species rich in these bioactive compounds mainly originate from South America and Asia, though some also come from Russia and Africa, where they have been traditionally used for centuries [20,21]. Most of these substances produce stimulating and/or hallucinogenic effects, while a few exhibit relaxing and/or sedative properties [19,21].

Classifying the plant-based smart drugs currently in circulation is particularly challenging: some researchers categorize them based on their method of consumption, others by the chemical classes of the bioactive compounds they contain, and still others according to their intended use [15]. In this review, they have been categorized based on the bioactive substances they contain.

#### 3.1.1. Alkaloid-Containing Plants

##### Ergoline Alkaloids

Among the plants containing ergoline alkaloids, the most widely known are *Argyreia nervosa* (Burm.f.) Bojer, *Ipomoea violacea* L., and *Ipomoea corymbosa* Roth. Their seeds are used to induce psychoactive effects like those of lysergic acid diethylamide (LSD) [20].

*A. nervosa*, commonly known as *Hawaiian Baby Woodrose*, *Adhoguda*, *Vidhara*, *Elephant Creeper*, and *Woolly Morning Glory* [21,22], is a perennial climbing plant with pink flowers, native to India and widely distributed across Africa, Europe, and subtropical regions of the Americas [19]. In traditional Indian Ayurvedic medicine, the entire plant has been used for centuries to treat various ailments, including bronchitis, tuberculosis, arthritis, diabetes, and nervous disorders [20]. The leaves exhibit antimicrobial activity and have been used as local stimulants, rubefacients, and vesicants. Its roots are still used today for their antirheumatic properties and in the treatment of gonorrhoea, peptic ulcers, and nervous system diseases [15,20]. Additionally, the plant is used as a tonic, diuretic, and aphrodisiac, with studies highlighting its hypoglycaemic, hepatoprotective, immunoregulatory, analgesic, and anti-inflammatory properties [19,20,23]. While no traditional uses are recognized in Western countries, *A. nervosa* has recently drawn attention due to increasing reports of its abuse as a psychedelic drug [22].

*I. violacea*, also known as *Morning Glory*, *Heavenly Blue*, *Pearly Gates*, *Flying Saucers*, *Blue Star*, *Wedding Bells*, *Summer Skies*, or *Badoh Negro*, is a perennial climbing plant native to South America. Its seeds, known as *tlitliltzin* by the Aztecs, were traditionally used in divination ceremonies by Native American cultures for their hallucinogenic properties [15,20].

*I. corymbosa*, another perennial climbing plant with white flowers, is commonly referred to as *Christmasvine*, *Christmaspops*, or *Snakeplant*. Native to Latin America, it has since spread to other regions [20]. Its seeds, known as *Ololiuqui* in Aztec culture, have a long history of use in Central Mexico [15]. The plant has also been employed in traditional medicine, and according to some sources, it may have played an even more significant role in religious rituals than hallucinogenic mushrooms or peyote [15,20]. Even today, certain isolated tribes in the remote mountains of Southern Mexico continue to use *I. corymbosa* seeds in spiritual practices [15].

The psychoactive alkaloids responsible for the hallucinogenic effects of these plants are found exclusively in their seeds. The primary compounds include ergine, also known as lysergamide, lysergic acid amide (LSA), and isoergine [21]. However, additional alkaloids such as erginine, ergometrine, lysergol, elimoclavine, chanoclavin I, chanoclavin II, peniclavine, ergometrinine, and ergine have also been isolated from these plants, though their specific effects remain largely unknown [19].

In comparison to *I. corymbosa* seeds, the total alkaloid content in *I. violacea* seeds is approximately five times higher. However, both species contain significantly lower concentrations than *A. nervosa*, which seeds have ten times the alkaloid content of *I. violacea*. As a result, only 5–10 seeds of *A. nervosa* are typically needed to achieve psychoactive effects, whereas 100–300 seeds are required for the other two species [15].

##### Pseudoalkaloids

Particularly popular among plant-based smart drug users are *Ephedra sinica* Stapf and *Sida cordifolia* L., which are rich in the pseudoalkaloid ephedrine and are therefore sold as central nervous system (CNS) stimulants and euphorizers [15].

*E. sinica*, also known as *Ma-huang* (a Chinese term specifically referring to the aerial parts of the plant), is a shrub of Chinese origin that has been known and used for over 5000 years [24,25]. The aerial parts of *Ephedra* sp. contain variable percentages of alkaloids, which are primarily concentrated in the stem internodes [15] and are responsible for its pharmacological effects [25]. In addition to ephedrine (the main bioactive constituent), other alkaloids such as d-pseudoephedrine, norephedrine, norpseudoephedrine, N-methylephedrine, and N-methylpseudoephedrine [25,26] have been identified. Traditional Chinese medicine recognizes the healing properties of the dried plant stems, which are administered as an infusion [15]. For thousands of years, the plant has been used in China for the treatment of asthma. However, its more recent use is in the form of dietary supplements marketed with claims to enhance energy and alertness, improve athletic performance, or promote weight loss [26].

These supplements are often combined with other products containing natural sources of caffeine to potentiate the effects of ephedrine, creating a combination of substances referred to as “stimulants”, which are used recreationally, for example, in nightclubs. Another significant issue is the heterogeneity of *Ephedra*-based products sold in smartshops, both in terms of bioactive compound content and in their combination with other plant extracts containing pharmacologically active molecules. These products are generally known as “herbal ecstasy” [15].

*S. cordifolia* is a perennial shrub belonging to the *Malvaceae* family, which seeds, leaves, and roots contain ephedrine, along with other bioactive compounds such as pseudoephedrine, vasicinone, and vasicine [27,28,29]. The amounts of ephedrine and pseudoephedrine found in *S. cordifolia* leaves are lower (less than 2%) compared to those in *E. sinica*. The roots, leaves, seeds, and stems of this plant have a long history of use in traditional Indian medicine for the treatment of various ailments, including asthmatic bronchitis, nasal congestion, colds, flu, respiratory failure, headaches, cough, edema, and osteoarticular pain [28,30,31]. According to Ayurvedic medicine, in addition to its effectiveness in treating respiratory system disorders, the plant also possesses tonic, astringent, emollient, and aphrodisiac properties [29,31]. Even today, *S. cordifolia* is used in Ayurveda as an adjunct therapy for asthma and is often mixed with other herbs to enhance vital energy and body tone. The *S. cordifolia* extract, which can contain between 0.8% and 1.2% ephedrine and can stimulate the CNS with effects like those of amphetamine, is readily available on specialized websites. It is often sold and sought after by smart drug users for its euphoric effects [15].

##### Tropane Alkaloids

Plants belonging to the *Solanaceae* family, such as *Datura stramonium* L. and *Brugmansia arborea* (L.) Steud., are highly popular among smart drug users for their hallucinogenic effects due to the presence of tropane alkaloids such as atropine, scopolamine, and hyoscyamine [26].

*D. stramonium*, native to the USA, is also known as *Jimson Weed*, *Locoweed*, *Thorn Apple*, *Devil’s Trumpet*, *Stink Weed*, and *Jamestown Weed* [20,26]. Although all parts of the plant are toxic, the seeds contain the highest concentration of tropane alkaloids—100 seeds can contain up to 6 mg of atropine—approximately ten times the amount found in the leaves [8,19]. Traditionally, the plant was used by Native Americans for medicinal, mystical, and religious purposes. Furthermore, in Western medicine, *D. stramonium* was historically used to treat asthma [19,20,26].

Today, adolescents seeking intense sensations and hallucinogenic effects most commonly use *D. stramonium* recreationally by ingesting the seeds or flowers whole; preparing an infusion from the leaves and crushed seeds; or smoking dried and crushed flowers, leaves, and seeds [19,20,26]. Euphoria, surreal interactions with the world, and visual hallucinations are among the most frequently reported effects of *D. stramonium* use [20].

*B. arborea*, commonly known as *Angel’s Trumpet*, is a shrub native to South America and widely cultivated as an ornamental species in Europe, the Southeastern USA, Australia, and Asia [32,33,34]. Tropane alkaloids are present throughout the plant but are primarily concentrated in the roots and seeds [35]. Native Americans historically used this plant as a hallucinogenic drug in ritual ceremonies, as a poison, in funeral rites, and for medicinal purposes [33]. In Peru, beyond its ritual use, the plant was valued for its analgesic, anti-inflammatory, vulnerary, decongestant, antispasmodic, and anti-rheumatic properties [32,34].

Due to its easy and inexpensive availability in gardens, homes, and online markets, interest in *Angel’s Trumpet* has increased over the years, particularly among teenagers and young adults who use it as a legal hallucinogen. This rise in use has been accompanied by an increasing number of intoxications recorded among smart drug users [36,37]. Göpel and colleagues [37] reported three cases of psychosis induced by *Angel’s Trumpet* tea in drug-addicted adolescents. As previously noted for *D. stramonium*, recreational drug users often consume *B. arborea* by ingesting the intact flowers; smoking the dried leaves; or preparing an infusion with flowers, leaves, or seeds [36].

##### Indole Alkaloids

*Mitragyna speciosa* (*Rubiaceae*) is a tropical evergreen tree native to Southeast Asia, the Philippines, and New Guinea, now also distributed in other regions of the world [19,38]. It is commonly known as *kratom*, a term that refers both to the plant itself and to the botanical products derived from its leaves [39]. In Southeast Asian countries, especially Thailand and Malaysia, *M. speciosa* has been used for centuries for its stimulant and narcotic properties, as a recreational drug, pain reliever, and for the treatment of diarrhea and opiate addiction [20,40]. The effects of *kratom* are dose-dependent; at low doses (1–5 g), it exhibits stimulating effects, while, at higher doses (5–15 g), it induces sedative/narcotic opioid-like effects [20,40]. The plant has been used since ancient times by Asian farmers to counteract fatigue and improve work productivity [19,39] and by Thai natives for its opioid- and cocaine-like effects [19,20,39]. Recently, *M. speciosa* has begun to be consumed recreationally in Europe and the USA [19,39]. Traditionally, *kratom* was consumed by chewing fresh, midrib-less leaves. Dried leaves can also be chewed, but more commonly, after being chopped or pulverized, they were smoked or infused [19,40]. Both fresh and dried leaves can be boiled for an extended period to prepare a paste-like extract that can be stored for a long time [20]. Currently, dried and powdered leaves are consumed recreationally by those seeking legal substances with stimulating properties or sedative effects [15]. Various formulations of *M. speciosa*, such as raw leaves; powders; beverages; and new preparations like capsules, resins, or tinctures, are now available online and can be easily purchased, making consumption more accessible [19,21]. More than 40 alkaloids have been identified in *kratom* [41]. Among these, only four are known to be pharmacologically active: mitragynine, which is exclusive to *M. speciosa* and is the most abundant psychoactive compound in the plant (up to 66% of total alkaloids); 7-hydroxymitragynine; speciociliatine; and corynantheidine [39], all indole alkaloids [38]. Other alkaloids present in significant concentrations, such as speciogynine, paynantheine, and mitraphylline, along with those found in trace amounts, are not known to be pharmacologically active but may contribute synergistically to the overall effects of *kratom* [39]. Currently, *M. speciosa* is not illegal in most European countries or the USA [41], but it is strictly controlled in several countries, including Denmark, Latvia, Lithuania, Poland, Romania, and Sweden, due to its high potential for misuse. In Australia, Malaysia, Myanmar, and Thailand, *kratom* is regulated under the Narcotics Act, while, in New Zealand, both the plant and mitragynine are controlled by the Medicines Amendment Regulations [21,41]. The Drug Enforcement Administration (DEA) has placed *kratom* on its list of “drugs of concern” [21].

*Pausinystalia johimbe* (K. Schum.) Pierre, more commonly known as *yohimbe*, is an evergreen tree belonging to the *Rubiaceae* family, primarily found in tropical West Africa [40]. The bioactive compounds present in its stem bark are indole alkaloids, the most abundant of which is yohimbine (10–15% of the total content), followed by its stereoisomers rauwolscine (*α*-yohimbine), *β*-yohimbine, *ψ*-yohimbine, corynanthine, corynantheine, allo-yohimbine, and yohimbic acid [21,40]. Traditionally, the bark is used as an aphrodisiac for the treatment of erectile dysfunction [21], and for this reason, its use has rapidly spread to Western countries [15]. Generally, the pulverized or ground bark is consumed as a decoction with other herbs, but it is also smoked or snorted for its hallucinogenic properties. In online smartshops, *yohimbe* is also available in capsule form, often combined with other herbs [15].

*Sceletium tortuosum* (L.) N.E.Br., commonly known as *Kanna*, *Channa*, or *Kougoed*, is a creeping perennial plant with succulent leaves belonging to the *Aizoaceae* family [40,42,43]. It is native to South Africa, where it has been traditionally used by the Khoikhoi and San tribes to relieve thirst and hunger; combat fatigue; elevate mood; and for healing, social, and spiritual purposes [21,40,42]. The dried aerial parts are commonly chewed or consumed as infusions, decoctions, and tinctures and are sometimes smoked or used as snuff [40]. Today, *S. tortuosum* is sold on various websites in the form of capsules or tablets and is recommended for the treatment of depression and anxiety, as an aid for smoking cessation, for attention deficit disorders, and as a cognitive enhancer during intense study periods, as well as for improving social interactions and increasing sexual desire [21]. When snorted, significant effects occur at doses as low as 20 mg, and when consumed in combination with *Cannabis* or alcohol, intense hallucinatory effects can be experienced [15]. Mesembrine-like indole alkaloids, including mesembrine, mesembrenol, mesembranol, mesembrenone, and tortuosamine, are the primary bioactive compounds responsible for the psychoactive and stimulant properties of *S. tortuosum* [43].

*Voacanga africana* Stapf (*Apocynaceae*) is a small flowering tree native to tropical and subtropical forests of West Africa [44]. The plant contains a series of ibogaine-type indole alkaloids, including ibogaine and tabersonine (the most abundant), voacamine, and voacangine [15,40]. Other alkaloids recently isolated from this plant include voacandimine, 3,6-oxovoacangine, 5-hydroxy-3,6-oxovoacangine, and voacangalactone [45]. The alkaloid content varies within the plant: 5–10% in the root, 4–5% in the stem bark, 0.3–0.45% in the leaves, and 1.5% in the seeds [15]. *V. africana* has been known and used in Africa since ancient times [44] for religious purposes and in traditional medicine for treating infectious diseases, mental disorders, and pain relief [15]. On the Ivory Coast, Ghana, Cameroon, and the Congo, the stem bark is used to treat leprosy, diarrhoea, ulcers, convulsions in children, generalized edema, and microbial infections. In Cameroon, extracts from the fruit, leaves, and seeds are used to treat orchitis, gonorrhea, and dental caries, respectively [44,46]. West African shamans ingest the bark and seeds as psychostimulants for divination [15]. Today, *V. africana* is sold for recreational use in online and physical smartshops, where its seeds and bark are purchased for their alleged hallucinogenic, aphrodisiac, and psychoactive properties [15,45].

##### Other Alkaloids

*Areca catechu* L. is a palm belonging to the *Arecaceae* family, native to Sri Lanka and Malaysia and now widely distributed across Asia, Africa, Europe, and the Americas [21]. The fruit of this palm, known as the areca nut or more commonly as the *betel nut*, has been consumed for centuries in traditional Southeast Asian medicine and rituals [19,21]. Its consumption has been associated with a sense of well-being, psychostimulant effects, stress reduction, breath freshening, anthelmintic properties, and appetite stimulation [19,47]. However, it has also been used in the treatment of various conditions, including malaria, fever, hernia, hypertension, urinary stones, digestive disorders, and diarrhea [19]. Beyond its medicinal use, the widespread abuse of this fruit for its relaxing, stimulating, and aphrodisiac effects has made it one of the most widely consumed addictive substances globally. It ranks as the fourth most used psychoactive substance, following nicotine, ethanol, and caffeine [19,40], with an estimated 600 million people worldwide consuming *betel nut*. The nut is consumed both alone and in combination with other substances in the form of *betel quid*, a preparation consisting of ground areca nut mixed with calcium hydroxide (lime) and *Piper betel* leaves (*betel pepper*), which is chewed or kept in the mouth to slowly release its active compounds [15,21]. Additionally, the nut can also be smoked, mixed with tobacco, and wrapped in *Piper betel* leaves [15].

To date, more than 59 molecules belonging to different chemical classes have been identified in this fruit, including alkaloids, tannins, flavonoids, triterpenoids, steroids, and fatty acids [21]. Although several alkaloids such as arecaidine, arecolidine, guvacoline, and guvacine have been identified in the *A. catechu* nut, the main psychoactive compound is arecoline, a pyridine alkaloid [19]. Currently, there are no restrictions on the use of this plant or its bioactive compounds in Europe or the USA [21].

*Eurycoma longifolia* Jack (*Simaroubaceae*), commonly known as *tongkat ali*, is a flowering plant native to Southeast Asian countries, including Indonesia, Malaysia, Vietnam, Cambodia, Myanmar, Laos, and Thailand [48,49]. In these regions, a decoction of the root has been used for centuries in traditional medicine to treat sexual dysfunction, enhance male libido, and increase testosterone levels, as well as to address a variety of ailments, including malaria, fever, hypertension, cancer, fatigue, psychophysical stress, diabetes, anxiety, constipation, osteoporosis, syphilis, and glandular swelling [48,49]. Among the various bioactive compounds isolated from the roots of this plant, the most abundant include the alkaloid canthin-6-one, the quassinoids eurycolactone A–E, euricomalactone, euricomanone, euricomanol, and lauricolactone A and B, as well as β-carboline derivatives [48,50]. Currently, *E. longifolia*-based extracts are widely sold online as dietary supplements intended to enhance sexual desire, boost testosterone levels, improve athletic performance, and aid in weight loss [15].

*Heimia salicifolia* (Kunth) Link. (*Lythraceae*), a plant known for its alleged psychoactive properties, is among the thirty most used substances for recreational purposes, according to studies conducted on major websites frequented by psychotropic substance users in Europe [51]. Phytochemical analysis of *H. salicifolia* leaves has revealed the presence of multiple quinolizidine alkaloids, including vertine (cryogenine), heimidine, lythrine, nesodine, lyfoline, and dehydrodecodine, which concentrations vary significantly [15,52]. Among these, vertine is the most abundant and is considered the primary compound responsible for the plant’s pharmacological effects [53]. *H. salicifolia* is a shrub found mainly in Mexico, Texas, El Salvador, Jamaica, and South America [52,53]. Depending on the region, it is known by various names, such as *sinicuichi* in Mexico and *abre-o-sol* in Brazil [52]. Considered sacred by the Aztecs, this plant was traditionally consumed during shamanic rituals. Today, infusions of its leaves are still used by indigenous peoples of Central and South America for their antisyphilitic, antipyretic, emetic, hemostatic, tonic, laxative, diuretic, anti-inflammatory, digestive, and wound-healing properties [52,53]. A beverage made from *H. salicifolia* leaves is believed to induce exhilaration, a state of inebriation, muscle relaxation, and a pleasant drowsiness, as well as auditory distortions and a yellow-tinted vision [52,53].

Finally, *Trichocereus* species, known for their high concentration of the psychoactive alkaloid mescaline, are also widely sought after in the recreational domain for their psychedelic properties [20]. These cacti, including *T. pachanoi*, *T. peruvianus*, *T. werdermannianus*, *T. macrogonus*, and *T. validus*, are native to the Andean region, which stretches from Ecuador through Peru, Bolivia, Chile, and Argentina, extending to the Atlantic coast. Due to their hallucinogenic properties, these cacti have long been used by Andean shamans in religious and healing rituals [15].

#### 3.1.2. N,N-Dimethyltryptamine

Among the plants consumed for recreational purposes to achieve hallucinogenic effects, there are also species such as *Psychotria viridis* Ruiz & Pav., *Mimosa tenuiflora* (Willd.) Poir., and *Anadenanthera peregrina* (L.) Speg., which are characterized by the presence of N,N-dimethyltryptamine (DMT). This bioactive compound belongs to the tryptamine class and shares, in terms of intensity and characteristics, psychedelic and hallucinogenic properties with LSD and mescaline [54,55].

*P. viridis*, a shrub of the *Rubiaceae* family, has DMT-rich leaves that are often combined with the stem bark of the liana *Banisteriopsis caapi* Spruce ex Griseb. (*Malpighiaceae*) and other plants [54,55,56] to prepare a well-known psychoactive drink called *ayahuasca*, a Quechua term meaning “vine of souls” [19,56,57,58]. *Ayahuasca* is also known by various other names, including *hoasca*, *yagé*, *daime*, *caapi*, and *natema*, depending on the region of Colombia, Ecuador, Brazil, Bolivia, and Peru. It is a traditional entheogenic decoction that has been consumed for centuries, originally by indigenous peoples of the Amazon River basin, for ritual and religious purposes, magical experiences, and healing [58,59]. Recently, its use has spread to Europe and North America for recreational purposes [59], facilitated by online platforms and smartshops that have contributed to the popularization of this psychoactive drink [56].

*B. caapi* does not contain DMT but is rich in β-carboline alkaloids, including harmine, harmaline, and tetrahydroharmine [55]. The synergistic interaction between the alkaloids of *B. caapi* and the DMT from *P. viridis* appears to be responsible for the psychotropic effects of *ayahuasca* [19]. In fact, DMT from *P. viridis* is inactive when orally ingested, as it is rapidly metabolized by the liver and intestinal monoamine oxidase (MAO) [19,55]. However, when ingested together with β-carboline alkaloids, which act as reversible MAO inhibitors, it reaches the central nervous system without being degraded [56,59]. Currently, the regulation of *ayahuasca* remains controversial [21]. In 1970, DMT was classified as a Schedule I substance under the US Controlled Substances Act [59]. While products containing DMT are classified as prohibited substances not only in the USA but also in some European countries such as Italy, the consumption of *B. caapi* and *P. viridis*, or parts of them, is not regulated. Furthermore, the specific use of *ayahuasca* is currently legal in Brazil and the USA for religious rituals [21].

DMT was first isolated from the root bark of *M. tenuiflora* (*Fabaceae*), also known as *Mimosa hostilis* (C. Mart.) Benth [60]. The bark of this small tree is traditionally used together with the seeds of *Peganum harmala* L. to prepare *jurema* or *jurema wine*, a visionary-hallucinogenic drink consumed for ritual purposes in African and Brazilian traditions. Like *ayahuasca*, *jurema wine* contains DMT, along with monoamine oxidase inhibitors (MAOIs) present in *P. harmala* seeds. Additionally, the bark of *M. tenuiflora* has been used by local populations in Central America to treat burns and skin wounds [15]. Today, various medicinal and cosmetic products derived from *M. tenuiflora* are available, particularly on the Mexican market, though their use remains largely empirical and rooted in traditional knowledge [61]. For recreational purposes, online smartshops often offer this plant in combination with other herbs containing MAOIs [15].

*A. peregrina* (*Mimosaceae*) is a tree native to the Caribbean and the open plains of South America. Its leaves, bark, and seeds contain hallucinogenic tryptamines, including bufotenin (5-hydroxy-N,N-dimethyltryptamine, 5-OH-DMT), which is the most abundant, followed by 5-methoxy-N,N-dimethyltryptamine (5-MeO-DMT) and DMT [62]. The seeds of *A. peregrina* are typically roasted and ground to prepare *yopo*, a snuff-like powder that is inhaled for its hallucinogenic effects by indigenous peoples of Southeastern Venezuela, Colombia, and Brazil [15,62,63]. The use of inhaled powders derived from *A. peregrina* is ancient and was widespread throughout Latin America [15]. Christopher Columbus’ sailors were among the first Europeans to document the use of *cohoba* in Brazil, a psychoactive mixture prepared from *A. peregrina*, *Nicotiana tabacum* L., and other plants. This blend was inhaled by shamans for its stimulating and hallucinogenic effects [64]. Even today, *yopo* is sniffed using bamboo tubes or bird bones by certain Venezuelan ethnic groups in the Orinoco Valley during shamanic rituals. Additionally, some Brazilian and Venezuelan tribes still consume *yopo* together with *B. caapi* to intensify and prolong the visionary effects, creating an experience like that of *ayahuasca* [63,65].

In online smartshops, *A. peregrina* seeds are categorized as ethnobotanical or horticultural products labeled as “not for human consumption”. However, these websites provide extensive information on the tryptamine content of the seeds, their effects, and even instructions on how to prepare the inhalable powder [15].

#### 3.1.3. Terpenes

*Artemisia absinthium* L., commonly known as wormwood, is a small shrub native to Europe, Asia, the Middle East, and North Africa and has also been naturalized in the USA [26,66]. It is well known for its traditional use in treating digestive and liver disorders, as well as for its antimicrobial, antihypertensive, antimalarial, antipyretic, and diuretic properties. This plant contains several bioactive compounds, including terpenes, polyphenols, and sterols, which are particularly abundant in its leaves, stems, and flowering tops [66]. The leaves and flowers of *A. absinthium* are key ingredients in the alcoholic beverage known as *absinthe*, which gained immense popularity in 19th century Europe and has recently seen a resurgence of interest [67], largely due to its alleged hallucinogenic effects. These effects are attributed to the presence of absinthine, a dimeric guaianolide belonging to the triterpene family, which is the primary bioactive compound in *A. absinthium* and is responsible for its distinctive bitter taste.

### 3.2. Synthetic Cannabinoids

Since the discovery of Δ^9^-tetrahydrocannabinol (Δ^9^-THC), the psychoactive compound in *Cannabis*, the pharmaceutical industry has conducted extensive research to develop synthetic analogs that could be used as research tools to explore the endocannabinoid system. The goal was to create compounds with the same biological activity as natural cannabinoids but without their psychoactive side effects [68,69,70,71].

These new molecules, known as synthetic cannabinoids (SCs), are manmade compounds with mind-altering properties. They include not only substances structurally like already known phytocannabinoids but also chemically distinct compounds [70,71]. However, because of these studies, illicit chemists have exploited published scientific literature on SCs to synthesize large quantities of these substances for illegal commercial use [72,73].

These cannabinoids, synthesized in clandestine laboratories, first appeared in Europe in the early 2000s. Since they could be consumed as a legal alternative to *Cannabis*, their popularity spread rapidly [69]. It was only in late 2008 that they began to be identified in various herbal blends known as *Spice*. Following these reports, numerous countries worldwide implemented restrictions on the production and sale of SCs [15,74]. Today, SCs represent the largest group of new psychoactive substances (NPS) reported by the European Monitoring Centre for Drugs and Drug Addiction (EMCDDA) through the EU Early Warning System [75]. According to the most recent update, 224 SCs have appeared on the drug market since 2008, including 15 newly identified in 2021 [75]. SCs are produced in powder form and are mostly sprayed onto inert plant material after being dissolved in solvents such as acetone or ethanol. Once the solvent evaporates and the plant material is dried, the final product is crushed and packaged [70]. They are marketed in online smartshops, head shops, and convenience stores as “herbal incense” or “smoking herbal blends” under various brand names, including *Spice*, *K2*, *Black mamba*, *Zombie*, *Bombay blue*, and others. These products are labeled “not intended for human consumption” [69,72].

Structurally, synthetic cannabinoids encompass a vast and diverse family of molecules belonging to various chemical groups and subgroups. New substances from previously unknown chemical classes continuously emerge, making the list of SCs nearly endless [76]. Functionally, SCs mimic Δ^9^-THC, although only a few are structurally related to this bioactive compound [76,77]. The primary distinction lies in their increased biological activity [70].

In recent publications, SCs are increasingly referred to as “synthetic cannabinoid receptor agonists” (SCRAs) due to their ability to act as agonists of cannabinoid receptors 1 and 2 (CB1 and CB2, respectively), although additional mechanisms of action may also exist [68]. Overall, SCs primarily function as full agonists at CB1 and CB2 receptors, while only a few act as partial agonists [68]. Despite their chemical diversity, SCRAs share common characteristics such as lipophilicity, non-polarity, and the presence of 22–26 carbon atoms [15,70]. Another key feature is their alkyl side chain, which plays a crucial role in the compound’s biological activity. For optimal activity, this chain typically contains between four and nine saturated carbon atoms [15]. Based on the structural differences, lipophilicity, and binding affinity for cannabinoid receptors, SCs can be classified into seven main groups [78,79]: classical cannabinoids, non-classical cannabinoids, hybrid cannabinoids, aminoalkylindoles, aminoalkylindazoles, eicosanoids, and other synthetic cannabinoids.

The classical cannabinoids, characterized by a dibenzopyran ring, include compounds naturally occurring in *Cannabis*, such as nabilone, as well as synthetic analogs structurally related to Δ^9^-THC [70,78,80]. These were the first synthetic cannabinoids developed, beginning in the 1960s [70]. An example is HU-210, a potent CB1 and CB2 agonist synthesized in 1988 [69,80,81].

The non-classical cannabinoids, developed in the 1970s, consist of cyclohexylphenols with cannabinoid-like activity [15]. These include CP-47947, a much more potent compound than Δ^9^-THC, and its analogs [70,80,81].

Hybrid cannabinoids combine structural features of classical and non-classical cannabinoids [80]. A well-known example is AM-4030, a derivative of HU-210, characterized by the presence of a dibenzopyran ring [70].

The aminoalkylindoles form the largest class of SCs, originally synthesized in 1991 [80]. This class includes naphthoylindoles, naphthylmethylindoles, phenylacetylindoles, and benzoylindoles [15,80]. They exhibit cannabimimetic properties despite their structural differences from Δ^9^-THC and are the most detected SCs in herbal smoking blends, likely due to their ease of synthesis [70]. Over time, new chemical classes of SCs have emerged in response to global regulations banning earlier compounds. One such class, the indazole-derived synthetic cannabinoids, has recently appeared on the drug market [15]. The aminoalkylindazoles are a subcategory further divided into naphthoylindazoles (e.g., THJ-018 and THJ-2201) and indazole carboxamides, which contain an additional nitrogen atom in the indole ring and a carboxamide functional group instead of a ketone [79,80]. Examples include 5F-APINACA, ADB-FUBINACA, ADB-CHMINACA, and AB-CHMINACA, which were among the most frequently seized synthetic cannabinoids in 2015 [68].

Eicosanoids, another class of SCs, are synthetic analogs of endocannabinoids such as anandamide [70]. A notable example is AM-356 [79].

Finally, other synthetic cannabinoids include structurally diverse compounds that do not fit into the above categories, such as diarylpyrazoles, naphthoylpyrroles, naphthylmethylindenes, and gamma-carboline-based synthetic cannabinoids [79,80].

### 3.3. Synthetic Cathinones

Synthetic cathinones constitute the second-largest group of new psychoactive substances, among more than 884 NPSs identified in Europe by the EMCDDA [75,82]. These compounds derive from 2-amino-1-phenyl-1-propanone or cathinone, a natural alkaloid present in the leaves of *Catha edulis* (Vahl) Endl. (khat), similar in structure and action to amphetamine [82,83,84,85]. Chewing the leaves of khat, a shrub native to East Africa and the Arabian Peninsula, has been practiced for centuries by the populations of these regions due to their euphoric and psychostimulant effects [82,86,87]. Today, it is still a popular practice in countries such as Somalia, Yemen, Kenya, and Ethiopia [86]. Over the years, in parallel with the increase in migration, chewing of khat has spread in Europe, as well as in the USA [86,87].

Khat leaves contain more than 40 compounds, including alkaloids, flavonoids, amino acids, glycosides, sterols, vitamins, and minerals. In the early 1930s, cathine ((+)-norpseudoephedrine) was identified as the active ingredient of khat [83]. However, the activity of this substance was thought to be insufficient to be responsible for all the observed pharmacological effects [83,87]. It was not until the 1970s that cathinone was isolated from khat leaves and was found to be 7- to 10-fold more potent than cathine [82,86].

The chemical structure and synthesis of some synthetic cathinones have been known for many years but have been only recently abused [85]. The first synthetic cathinone to appear on the pharmaceutical market, methcathinone (α-methylamino-propiophenone or ephedrone (EPH)), was synthesized in 1928 in the USA [83,87]. It is a methylated analog of cathinone used in the former Soviet Union as an antidepressant drug in the 1930s and 1940s. A decade later, it was studied as an analeptic drug in the USA, although it never went into commercial medical production due to its strong addiction potential [85,86,87]. The first reports of methcathinone abuse began to emerge in the Soviet Union from the 1970s and in the USA, where this drug was known as “Jeff”, “Mulka”, and “Cat”, from the early 1990s [85,86].

Around the 1950s, other synthetic cathinones continued to appear as potential drugs, including diethylcathinone (amfepramone or diethylpropion), introduced in 1958 as an appetite suppressant, but reports of abuse began to emerge shortly thereafter, and 1-(4-methylphenyl)-2-pyrrolidin-1-ylpentan-1-one (pyrovalerone), synthesized in the 1960s for use in the treatment of chronic fatigue and as an anorectic and appetite suppressant drug but withdrawn from the market a few years after its discovery due to reports of abuse [86,87]. In the same years, other derivatives of the pyrrolidinophenone family, to which pyrovalerone belongs, were synthesized but without any clinical intent, such as 3,4-methylenedioxypyrovalerone (MDPV), synthesized in 1967 [83].

A more recent analog, 1-(1,3-benzodioxol-5-yl)-2-(methylamino)propan-1-one or methylone (3,4-methylenedioxy-N-methylcathinone or βk-MDMA), was developed in 1996 as a potential antidepressant and antiparkinsonian drug, but it was never marketed due to its psychostimulant properties similar to MDMA [83,85,88].

The use of synthetic cathinones as therapeutic drugs is limited, mainly due to adverse effects and their abuse and addiction potential. Conversely, they have drawn attention for recreational use [87].

Chemically, synthetic cathinones are β-keto phenethylamine derivatives [83]. Methamphetamine and MDMA, being structurally like amphetamine, are commonly called β-ketoamphetamines [82]. All synthetic cathinones are based on the basic structure of natural cathinone, consisting of a phenyl ring and an aminoalkyl chain with a carbonyl group in the β-position [82,89]. Cathinone derivatives can be synthesized by substitutions at several key positions, including the aromatic ring, alkyl side chain, and nitrogen atom in the amino group [87,89]. Substitutions can give rise to an infinite number of derivatives [88,90]. Based on the substitution model, synthetic cathinones can be divided into four main structural groups [83].

Currently, synthetic cathinones are widely used and abused worldwide due to their psychostimulant and hallucinogenic effects, which are like cocaine, MDMA, or other amphetamines, but at a much lower cost [83,85]. However, these synthetic compounds can be far more potent than the drugs they mimic, leading to an increased risk of overdose and death [83].

Synthetic cathinones are mainly marketed online and are also distributed through smartshops, street-level dealers, head shops, smoke shops, and gas stations [83,85]. Although they are widely distributed in Europe, the USA, and many other countries worldwide, most synthetic cathinones are produced in China and Southeast Asia [84,85].

These compounds are commonly sold as “fertilizers”, “research chemicals”, “plant foods”, or “bath salts” under attractive brand names such as “Bloom”, “Ivory Wave”, “Vanilla Sky”, or “White Lightning” and are intentionally labeled as “not for human consumption” or “for research purposes only” to circumvent legal restrictions [85,87,91,92]. Among the synthetic cathinones, the most widespread is 2-(methylamino)-1-(4-methylphenyl) propan-1-one, commonly known as mephedrone (4-methylmethcathinone, 4-methylephedrone, 4-MMC) [93,94]. Mephedrone was first synthesized in 1929, but reports of its abuse did not emerge until the early 2000s [85,95]. This cathinone, marketed under various street names such as “meow meow”, “bubbles”, “4-MMC”, “M-Cat”, and “MMCAT”, is available in the form of fine crystals, powder, and occasionally tablets or capsules [93].

Among the best-selling and most widely abused synthetic cathinones are 1-(1,3-benzodioxol-5-yl)-2-(methylamino) butan-1-one, also known as butylone (βk-MBDB), methylone, and MDPV [85].

Several factors have contributed to the widespread popularity of synthetic cathinones, including their psychostimulant effects, which are like amphetamines and cocaine, with the addition of hallucinogenic properties. Their easy availability, low cost, appealing brand names, and packaging have further increased their appeal. Additionally, new compounds can be legally sold until they are formally listed as prohibited substances, allowing traffickers to exploit legislative loopholes. Furthermore, the absence of rapid screening tests for these compounds in biological samples makes it difficult to detect their use [15,87].

Regarding routes of administration, synthetic cathinones are commonly snorted as a white powder or consumed as crystals but are also taken orally in the form of capsules, aqueous solutions, and tablets or through nasal insufflation. Consumers often engage in “keying” (snorting the powder using a key) and “bombing” (ingesting the powder wrapped in cigarette paper). Less commonly, these substances are administered rectally, intravenously, or intramuscularly [82,84]. Cases of “eyeballing”, where synthetic cathinones are applied directly into the eye, have also been reported [84].

### 3.4. Designer Drugs

The term designer drug was first coined in the 1980s by Dr. G. Henderson, a pharmacologist at the University of California, to describe new synthetic substances derived from clandestine modifications of well-known illicit psychotropic molecules. These substances, which were already subject to strict government regulations, were chemically altered to mimic their psychoactive effects while remaining outside legal control due to the structural modifications of the original compounds [54,96,97]. Over the years, designer drugs have gained increasing popularity among recreational drug users and have spread rapidly, largely facilitated by online sales platforms [15,98]. The internet plays a crucial role not only in the distribution of these substances but also in the acquisition and exchange of information related to their use [97]. The number of designer drugs available on the illicit market continues to grow, with usage patterns evolving each year in response to new trends and regulatory controls. As a result, regulating these emerging substances and staying updated on their pharmacological and toxicological effects remains a significant challenge [97,98]. Within the category of designer drugs, four primary chemical classes can be identified: phenethylamines, tryptamines, piperazines, and piperidines. These classes serve as reference frameworks for categorizing new substances that emerge in online marketplaces or are seized at border customs in various countries [15].

*Phenethylamines* comprise a large group of psychoactive molecules, which also includes well-known drugs of abuse such as amphetamine, methamphetamine, paramethoxymethamphetamine (PMMA), and MDMA [99,100]. These molecules are further divided into subgroups based on different substitutions on the aromatic ring, alkyl chain, and nitrogen atom of the *phenethylamine* core structure. These structural variations determine the compound’s properties through interactions with the dopaminergic, noradrenergic, and serotonergic systems. While most phenethylamines exhibit stimulant and euphoric effects, some possess entactogenic and psychedelic properties [99].

The “*2C*” phenethylamine series is characterized by the addition of methoxy groups at positions 2 and 5 of the aromatic ring, with the possibility of a halogen at position 4. This group includes compounds such as 4-bromo-2,5-dimethoxyphenethylamine (2C-B), 4-iodo-2,5-dimethoxyphenethylamine (2C-I), and 2,5-dimethoxy-4-(n)-propylthiophenethylamine (2C-T-7). The *2C* phenethylamines act on serotonin 5-HT2C receptors, producing hallucinogenic effects and psychotic reactions [101].

The “*D*” series, which includes 2,5-dimethoxy-4-bromoamphetamine (DOB) and 2,5-dimethoxy-4-iodoamphetamine (DOI), is structurally similar to the *2C* series but features a methyl group on the side chain at the alpha position of the amino group [99]. This substitution pattern enhances hallucinogenic properties through agonistic action on serotonin 5-HT2 receptors [98]. The stimulant and psychedelic effects of these substances typically take over an hour to manifest, which increases the risk of repeated administration. The effects are long-lasting and can be intense even at low doses of a few milligrams [15,98]. Notably, this group of substances is more likely to induce vasoconstriction compared to other phenethylamines, which may explain the significant morbidity and mortality associated with their use [98].

Since 2010, a new class of psychedelic compounds known as NBOMe has emerged on the illicit drug market. This series consists of analogs of the *2C* series containing an N-(2-methoxy)benzyl substituent. NBOMe compounds function as potent agonists of serotonin 5-HT2A and 5-HT2C receptors, exhibiting higher affinity for 5-HT2A receptors than the *2C* series while displaying lower affinity, potency, and efficacy at 5-HT2B receptors compared to 5-HT2A and 5-HT2C. The first known NBOMe compound used recreationally was 25I-NBOMe (2-(4-iodo-2,5-dimethoxyphenyl)-N-[(2-methoxyphenyl) methyl] ethanamine), followed by the synthesis of other potent compounds such as 25B-NBOMe and 25C-NBOMe [102]. The benzofuran series includes phenethylamine analogs in which the benzofuran substitution on the phenyl ring enhances the stimulant and entactogenic properties. Among these, 4-(2-aminopropyl) benzofuran (4-APB), 5-(2-aminopropyl)benzofuran (5-APB), and 6-(2-aminopropyl)benzofuran (6-APB) have gained notable popularity [15].

Natural tryptamines with hallucinogenic properties have long attracted the interest of drug developers, who have synthesized structural analogs by modifying their chemical structure to enhance or mimic these effects [54]. The primary structural feature of tryptamines is the presence of an indole ring connected to an amino group via a two-carbon side chain [60]. Modifications at positions 6 and 7 of the indole ring typically reduce hallucinogenic activity, whereas most structural changes occur at positions 4 and 5 [54,98]. These substances exhibit high affinity for serotonin 5-HT receptors, with their psychoactive effects, including auditory, visual, and sensory hallucinations; altered personality; and distorted perception of space and time, being predominantly mediated by 5-HT2A and 5-HT1A receptors [54,97]. Synthetic tryptamines are widely available through online sales and have gained significant popularity as cheaper alternatives to classic hallucinogens. However, limited information regarding their acute and long-term effects, toxicological risks, and potential interactions with other substances presents serious public health concerns [54]. Among the most commonly encountered synthetic tryptamines on the illicit drug market are di-isopropyltryptamine (DiTP) and 4-hydroxy-N,N-diisopropyltryptamine (4-OH-DiPT) [15].

Piperazines constitute a large class of synthetic compounds that, despite being marketed as “herbal”, “herbal-effect”, or “natural” preparations, have no natural origin [98,103]. These molecules share a piperazine core structure to which various chemical groups are attached [54]. Recreational use of piperazines was first reported in the late 1990s, and although these substances have been widely abused, government control measures have only been introduced recently [103]. The two major structural groups of *piperazines* include 1-benzylpiperazines (e.g., 1-benzylpiperazine [BZP]) and 1-phenylpiperazines (e.g., 1-(m-chlorophenyl) piperazine [mCPP] and 1-(m-trifluoromethylphenyl) piperazine [TFMPP]). The former exhibit sympathomimetic stimulant properties, while the latter function primarily as serotonin reuptake inhibitors with minimal dopaminergic effects [54,98,103]. These substances are commonly sold online or on the illicit market in capsules, tablets, powders, or liquid form and are sometimes adulterants in cocaine and MDMA [54]. Their effects are like MDMA and amphetamines, with additional hallucinogenic properties [103].

The members of the piperidine and pyrrolidine classes are derived from phenethylamines, differing in the substitution of the amino group with either a piperidine or pyrrolidine ring. Representative compounds of these two groups include deoxypipraprodol (2-diphenylmethylpiperidine, 2-DPMP) and diphenylprolinol (diphenyl-2-pyrrolidinyl-methanol, D2PM), both of which are sought after for their psychostimulant effects due to their ability to inhibit dopamine reuptake [98,104].

## 4. Smart Drugs Analysis

As previously mentioned, a wide variety of legal highs are now available online and in retail stores across many European countries [105]. These products come in various formulations, including pills, tablets, smoking blends, powders, extracts, liquids, orodispersible strips, and chewing gums [106]. Some are even more discreetly packaged and marketed as bath salts, potpourri, incense, plant-based foods, and other non-consumable items. Many of these herbal blends are promoted as legal alternatives to *Cannabis* and are composed of plants naturally rich in alkaloids or stimulants. However, some of these products also contain synthetic cannabinoids that are deliberately added [107,108]; in such cases, the plants merely serve as a carrier medium for these illicit chemicals, facilitating their transportation and sale [109].

Recently, several European countries, as well as the United States and Canada, have banned the use of these substances. However, the main challenge regarding these herbal blends is their identification. Most of these products do not provide an ingredient list, and their complex composition makes it difficult to determine whether they contain naturally toxic plant metabolites such as alkaloids, synthetic toxic compounds such as synthetic cannabinoids [110], or if they are simply edible or ornamental aromatic plants.

In this context, a multidisciplinary approach [111,112,113,114], integrating molecular identification and micro-morphological analysis, is essential as a preliminary step before conducting phytochemical characterization. This process, however, requires appropriate analytical methods and updated reference databases covering bioactive compounds of both natural and synthetic origin to ensure accurate identification.

### 4.1. Molecular Analysis

DNA-based plant identification relies on the unique nucleotide sequence specific to each living organism [115,116]. Certain regions of DNA are conserved within the same species but vary among different species. Therefore, DNA-based methods are fundamentally dependent on the identification of DNA regions suitable for distinguishing between species. To establish a reliable identification system, it is essential to collect not only multiple samples of the target species but also samples of closely related species. For each taxon, detailed records must include the species name, voucher number, and accession number corresponding to the DNA sequences of the analyzed markers [111,115,116]. The primary technology underpinning these methods is the polymerase chain reaction (PCR). Today, commercially available kits allow for the extraction of high-quality DNA, free from polysaccharides or other metabolites that might interfere with DNA amplification. This is typically followed by the fluorometric determination of DNA concentration [111] and subsequent identification steps. Various techniques can be applied, including direct sequencing and other methods better suited for routine analysis, such as amplification-refractory mutation system (ARMS), high-resolution melting (HRM), and loop-mediated isothermal amplification (LAMP) [115,116]. The DNA-based approach also necessitates the development of a system applicable to a broad range of plant species. This system relies on the “master mix” concept, which consists of a PCR mixture containing all necessary components except primers and the DNA to be analyzed. The reaction is standardized, and only species-specific primers and the DNA of the plant species of interest are added to the master mix. Since PCR is a highly sensitive technique capable of detecting minute quantities of a target sequence, it can also be adapted for semi-finished and finished products [115,116].

For accurate species-level identification, the most recommended genetic analysis method is DNA barcoding [115]. However, successful application of this technique requires the extraction of high-quality DNA, making it particularly effective for fresh samples or those that have not undergone intensive processing. This methodology allows for the identification of the species to which a product or production batch belongs [115]. On the other hand, DNA mini-barcoding is particularly useful when obtaining a sufficiently long DNA sequence for standard DNA barcoding is challenging. This applies to biological samples that have undergone extensive processing, such as processed plant products [116]. The DNA mini-barcoding approach enables species recognition through the analysis of a shorter DNA segment (100–200 base pairs), compared to the 600–900 base pairs required for DNA barcoding, while still being distinctive enough to uniquely identify the species. However, this methodology is less frequently used because it often requires optimization and validation for the identification of specific species [116]. Unlike the previous methods, DNA metabarcoding allows for the simultaneous identification of all plant species present within a single sample [117]. This technique is ideal for analyzing semi-finished or processed products containing multiple plant species, such as herbal mixtures of unknown origin [117]. DNA extraction and sequencing are followed by complex bioinformatics analyses, requiring well-structured and validated protocols to ensure accurate species identification [116].

To determine the composition of an herbal blend, the obtained sequences are typically first analyzed using dedicated software such as ClustalW 2.1 [118], which performs sequence alignments to determine the number of Molecular Operational Taxonomic Units (MOTUs). The resulting sequences are then compared with reference plant lists and public DNA databases, such as GenBank, using Basic Local Alignment Search Tool (BLAST) analysis [119]. Each MOTU is identified by the species name that shows the closest match (≥95%) following the Barcode of Life Database Identification System (BOLD-IDS) guidelines [120]. This approach to plant identification in herbal blends is technically complex, requiring longer processing times and higher costs. However, it represents a highly effective tool at the national level for screening newly available smart drugs. Additionally, it can aid in the development of micro-morphological and analytical techniques for the qualitative and quantitative determination of bioactive compounds. Moreover, studies have shown that herbal blend preparation does not interfere with the success of DNA fingerprinting [111]. Nonetheless, there are still several limitations to overcome, including the need for better-defined reference databases, the incomplete deposition of accessions, and the fact that some DNA barcode entries lack all necessary BOLD descriptors for accurate species identification [121].

### 4.2. Micromorpholgy

The identification of a plant species typically begins with a morphological analysis as the first step. Morphological features can be assessed at different levels. Macroscopic examination is suitable for describing entire plants and their various parts, whereas micro-morphological examination involves analyzing features that require a stereomicroscope, which provides magnifications of up to 40–50×. These methods are particularly useful for examining smaller plant structures, fragmented plant material, small flowers, and trichomes [122].

However, a detailed micro-morphological analysis must be conducted using light-transmission microscopy. This technique can be applied by simply preparing an aqueous suspension of the powdered plant material. In some cases, specific sample preparation is required, such as clearing processes with an aqueous solution of 5% sodium hypochlorite or chloral hydrate solution, as well as embedding, sectioning, staining, and other preparatory steps [122]. Light-transmission microscopy typically offers magnifications ranging from 100 to 400×, reaching 1000× with the use of an immersion objective. Additional microscopic techniques, including bright-field, dark-field, phase contrast, polarization, and differential interference contrast optics microscopy, enhance the visibility of specific anatomical structures [122]. These techniques are well suited for studying plant tissues, cell walls, idioblasts, concretions, trichomes, epidermal appendages, and cellular inclusions such as calcium oxalate crystals.

In contrast, the ultrastructure of plants and plant cells can only be examined using scanning electron microscopy (SEM) and transmission electron microscopy (TEM). Although these techniques are significantly more complex and costly, requiring specialized sample preparation and skilled personnel, they are particularly valuable when combined with X-ray Energy-Dispersive System (EDS) detectors. This combination allows the differentiation between organic and inorganic crystals, making it possible to identify the presence of crystalline materials in herbal blends, which may indicate the presence of synthetic cannabinoids [111].

All these micro-morphological techniques are essential for the accurate identification of plant material, particularly for identifying plant species within herbal mixtures, which are generally composed of dried, fragmented, or powdered material. A precise micro-morphological analysis based on anatomical and histological features is crucial for species identification, allowing comparisons with microscopy atlases and databases, as well as references from medicinal plant, spice, and palynological studies [111].

To ensure the reproducibility, documentation, and traceability of results—like the quality control procedures used for herbal drugs—the morphological evaluation and identification of plant material should follow a standardized procedure [122]. The fundamental steps to be followed include (i) depositing and identifying a sample of the evaluated plant material (voucher specimen); (ii) using a standardized worksheet for each specimen, recording the main morphological characteristics of the investigated species, along with details of the specimen being analyzed. This should include space for additional expert annotations; (iii) applying appropriate measurement scales according to the microscopic technique used for photographic documentation; (iv) specifying the type of sample preparation performed for microscopic analysis (e.g., embedding, sectioning, or staining); and (v) indicating the microscopic technique used, referring to the type of illumination (bright-field, dark-field, polarized light, etc.) and magnification applied [122].

This approach enables the creation of a comprehensive data sheet containing all relevant information on the analyzed plant material, facilitating faster, more efficient, and accurate identification.

### 4.3. Chemical Analysis

The analysis of smart drugs varies significantly depending on their composition, particularly in the case of herbal blends, as well as on the chemical structure and properties of their bioactive compounds. Whenever possible, official methods should be used. However, the chemical analysis of smart drugs or the phytochemical analysis required for identifying complex herbal blends often necessitates the development and validation of ad hoc analytical methods. The development of such methods generally involves three key steps: evaluating the problem and characterizing the analytical requirements, developing the analytical method, and validating the analytical method. The first two steps are relatively straightforward when the chemical structure of the bioactive compounds is already known. However, the process becomes much more complex when smart drugs are composed of complex herbal blends, where the presence of multiple bioactive compounds and the potential matrix effect can interfere with the identification of the characteristic substances within the plant species or herbal blends under investigation. For this reason, the validation of an analytical method is crucial. This process involves determining fundamental parameters such as robustness, specificity, limit of detection (LOD), and limit of quantification (LOQ), in accordance with official guidelines such as the IUPAC, ISO, AOAC, EURACHEM, and ICH [123,124,125,126,127]. The robustness of a method ensures that it remains unaffected by small, deliberate variations in operating conditions. Specificity refers to the ability of the method to accurately and selectively determine the analyte of interest in the presence of other chemical or biological matrix constituents. The limit of detection represents the lowest concentration at which the analyte can be detected but not necessarily quantified, while the limit of quantification corresponds to the lowest concentration at which the analyte can be reliably quantified. By applying validated analytical methods, it becomes possible to correctly identify plant material, distinguishing between the plant species of interest and other species within the same genus, as well as differentiating between genera. This approach also allows for the analysis of raw materials, semi-finished products, and finished products. In all cases, appropriate sample preparation is a necessary preliminary step, which can involve a simple extraction process or, more commonly, an extraction followed by a purification step to ensure accurate results.

Various extraction and analytical techniques are routinely used for determining the composition of plant-derived smart drugs (Table 1) and synthetic smart drugs (Table 2). The analysis process begins with the extraction of molecules of interest from samples, and the most employed extraction methods include liquid–liquid extraction (LLE) and solid-phase extraction (SPE).

These techniques are particularly suitable for extracting liquid and solid biological samples after they have been homogenized in a suitable buffer. The choice of extraction and elution solvents depends on the specific analyte being targeted for analysis.

According to the available literature, chromatographic methods are the most widely used analytical techniques today. Among these, reverse-phase high-performance liquid chromatography coupled with a diode array detector (RP-HPLC-DAD) is the most employed method, primarily due to its cost-effectiveness and versatility. In these analyses, C18 is the most frequently used stationary phase, while acidified water with acetonitrile or methanol is typically used as the elution solvent. Chromatographic methods coupled with mass spectrometry represent the second-most employed technique. Liquid chromatography-mass spectrometry (LC-MS or LC-MS/MS) is widely preferred, followed by gas chromatography-mass spectrometry (GC-MS), which is particularly useful for the determination of volatile compounds and is sometimes coupled with a flame ionization detector (GC-FID). In some cases, in addition to a classic autosampler, static or dynamic headspace sampling is used, proving especially valuable for analyzing volatile compounds from hydrophilic samples or biological matrices, where the compounds are simply dispersed in water before analysis.

## 5. Neuropharmacological Effects of Amphetamine-Based Smart Drugs

The increasing use of synthetic and natural smart drugs, also known as nootropics (a broader category of cognitive enhancers), to enhance cognitive functions has attracted significant interest while also raising concerns about their long-term neuropharmacological effects. These substances are known to elevate levels of neurotransmitters such as dopamine, norepinephrine, and, in some cases, serotonin, temporarily improving concentration, working memory, and mental stamina. However, their neuropharmacological effects are complex and potentially harmful [167].

Natural and synthetic smart drugs exert different neuropharmacological effects. Modafinil, for example, is a wakefulness-promoting agent that primarily modulates the dopamine system by inhibiting dopamine reuptake, thereby increasing its availability in the brain. This mechanism enhances alertness and the ability to sustain attention over prolonged periods [168]. However, this artificial elevation of dopamine levels can lead to neuroadaptive changes, such as the downregulation of dopamine receptors, potentially resulting in tolerance and addiction with chronic use [169]. Another synthetic smart drug, methylphenidate, commonly known by the brand name Ritalin, is a widely used nootropic that blocks dopamine and norepinephrine transporters, increasing their synaptic concentration. This results in temporary improvements in concentration and working memory. However, studies have shown that prolonged use of methylphenidate can alter neuroplasticity, potentially impairing the brain’s ability to adapt to new stimuli or recover from neuronal damage [170].

In contrast, natural origin smart drugs can influence brain chemistry in unpredictable ways. *E. sinica*, is an ancient herb used for thousands of years in traditional Chinese medicine to treat conditions such as asthma, bronchitis, and colds. The active alkaloids, primarily ephedrine and pseudoephedrine, act as potent sympathomimetic agents. While its traditional use is well documented, *E. sinica*’s classification as a nootropic presents both potential cognitive benefits and significant health risks. Ephedrine, the most potent compound in *E. sinica*, stimulates adrenergic receptors in the central and peripheral nervous systems, acting as a non-selective agonist that promotes the release of norepinephrine, dopamine, and, to a lesser extent, serotonin. This results in increased neuronal activity, heightened arousal, improved attention, and enhanced cognitive function [171]. However, *E. sinica* exhibits both cognitive-enhancing and potentially dangerous neuropharmacological effects. While its ability to boost focus, alertness, and short-term memory makes it appealing for cognitive enhancement, its profound cardiovascular risks, potential for abuse, and neurotoxic side effects outweigh its benefits. The nootropic effects of *E. sinica* should be approached with caution, and its use as a smart drug is generally not recommended due to the well-documented risks. Other compounds, such as those found in *Salvia divinorum*, can induce hallucinations or severe psychological effects [172]. Furthermore, interactions between natural nootropics and the brain’s neurotransmitter systems can be highly complex. For instance, *Rhodiola rosea*, known for its adaptogenic properties, may influence serotonin and dopamine levels, potentially affecting mood and cognitive function. However, these effects are not fully understood and may contribute to neurochemical imbalances or exacerbate preexisting neurological conditions. Prolonged overstimulation of these neurotransmitter systems can also lead to neurotoxic effects. Sustained dopaminergic stimulation, for example, has been linked to oxidative stress and excitotoxicity in the prefrontal cortex, potentially causing neuronal damage and impairing neuroplasticity—the brain’s ability to reorganize and form new neural connections in response to learning and experience [173]. This neurotoxicity may manifest as long-term cognitive deficits, particularly in tasks requiring executive function and working memory, ultimately counteracting the initial cognitive benefits provided by these drugs.

The alteration of neuroplasticity could also impair the brain’s natural ability to recover from injuries or adapt to new experiences, potentially leading to a decline in cognitive resilience [174]. Additionally, the impact of smart drugs extends to other neurotransmitter systems. For example, modafinil’s effect on the histaminergic and orexinergic systems, which regulate wakefulness and arousal, further contributes to its stimulant properties. However, chronic manipulation of these systems may disrupt natural sleep–wake cycles and other homeostatic processes, leading to sleep disorders and neuropsychiatric complications [175].

In summary, while smart drugs such as modafinil and methylphenidate, as well as *Ginkgo biloba* and other plants used either alone or in herbal blends, can enhance cognitive performance through complex neuropharmacological mechanisms, their long-term use carries significant risks. These include neuroadaptive changes that reduce drug efficacy, potential neurotoxicity leading to cognitive decline, and disruptions to the brain’s natural plasticity, which may ultimately outweigh their initial cognitive benefits.

## 6. Psychological and Adverse Effects of Synthetic Cannabinoids, Cathinones, and DMT-Based Substances

Nootropics are a class of substances reputed to enhance cognitive functions such as memory, concentration, creativity, and overall mental performance. These substances range from prescription medications like modafinil and Adderall (methylphenidate) to natural supplements such as *G. biloba* and caffeine. Although they are increasingly popular among students and professionals seeking a mental edge, the use of smart drugs is accompanied by significant adverse effects and health risks, particularly when used without medical supervision.

Prescription stimulants like Adderall, which is composed of mixed amphetamine salts, are commonly used off-label for cognitive enhancement. While Adderall can improve attention and focus in the short term, its use is associated with several adverse effects, including increased heart rate, elevated blood pressure, and heightened cardiovascular risks. Long-term use can lead to dependency and addiction due to its stimulating effects on the central nervous system, which mirror those of more illicit stimulants. Additionally, psychiatric symptoms such as anxiety, paranoia, and even psychosis have been reported, particularly in individuals predisposed to mental health disorders [176].

Modafinil, another popular smart drug originally developed to treat narcolepsy, is often touted as a safer alternative to traditional stimulants. However, its use is not without risks. Common side effects of modafinil include headaches, nausea, nervousness, and dizziness. More severe adverse reactions, though rare, can include serious skin rashes such as Stevens–Johnson syndrome and psychiatric symptoms like anxiety and hallucinations. Its impact on sleep architecture is also concerning, as it can disrupt normal sleep patterns, leading to reduced sleep quality and potential long-term consequences for brain health [177].

Beyond these pharmaceutical options, even natural nootropics and over-the-counter supplements carry risks. Caffeine, one of the most widely consumed cognitive enhancers, can cause anxiety, restlessness, insomnia, and, in high doses, cardiovascular issues. Chronic overconsumption of caffeine has also been linked to tolerance and withdrawal symptoms, such as headaches and irritability, which can negatively impact mental performance and overall well-being [178]. Similarly, the use of *E. sinica* as a cognitive enhancer raises concerns. Its addictive potential and serious health risks make it a questionable option, particularly when safer alternatives are available. The line between medical use and abuse becomes blurred, especially in populations seeking rapid cognitive or physical stimulation without understanding the long-term health implications.

Overall, while natural nootropics offer potential cognitive benefits, their use should be approached with caution. The risks associated with their adverse effects, potential interactions with other medications, and the lack of long-term safety data highlight the importance of using these substances under the guidance of a healthcare provider. Just like synthetic nootropics, more research is needed to fully understand the long-term effects and safety profiles of natural cognitive enhancers. Moreover, the long-term effects of many nootropics remain poorly investigated. This lack of comprehensive research raises significant concerns about potential neurotoxicity and the long-term mental health implications of chronic use. For example, while short-term studies may show cognitive benefits, there are insufficient data on whether prolonged use could lead to cognitive decline, emotional disturbances, or other neurological issues. The complex interactions between these drugs and the brain’s natural neurochemical balance further complicate the risk profile, particularly in the absence of medical guidance [179]. To clearly summarize the psychological and adverse effects discussed throughout this review, Table 3 provides a comprehensive overview.

Significant concerns regarding adverse effects also arise from documented cases of fatal overdoses related to new psychoactive substances. It is important to underline that official reports published by the EMCDDA have documented multiple cases of fatal overdoses linked to the consumption of various novel psychoactive substances. Synthetic cannabinoids and synthetic cathinones (commonly known as “bath salts”) are among the most frequently cited drug classes in these reports, reflecting their high risk of toxicity and lethality. Epidemiological studies further confirm a rising incidence of emergency interventions and fatalities directly related to these compounds, emphasizing the urgent public health challenge posed by their widespread availability and misuse [75,191]. Therefore, continuous monitoring, timely reporting, and public health awareness campaigns remain essential components of an effective response to mitigate these risks.

## 7. The Clinical Impact of Cognitive and Mood Enhancers

Historically, the study of cognitive enhancers originates from the work of neurologist S. Freud, who, in 1885, published *Über Coca*, a scientific study in which his interest in cocaine emerged from the observation that medicine abounded in drugs that inhibited the nervous system but lacked substances capable of enhancing its performance. One of the applications Freud envisioned for this substance, in addition to its use as a local anesthetic, was in cases of neurasthenia, where symptoms of fatigue, easy exhaustion, and depressed mood appeared. Based on experiments conducted on both healthy subjects and him, Freud stated that, within minutes of intake, there was a sensation of euphoria, an increase in self-control, and an enhanced sense of vigor, along with an increased capacity for work. According to his observations, prolonged and intense mental or physical labor could be performed without any sensation of fatigue, as if the need for food and sleep had been eliminated, without causing any disturbances [192].

More recently, the scientific community has renewed its interest in understanding the effects of substances that stimulate the central nervous system (CNS), particularly in relation to a global social phenomenon: the increasing use and abuse of cognitive enhancers. These substances, which have not been historically recognized among traditional drugs of abuse, are not classified as prohibited narcotic or psychotropic substances. Their sale is not restricted, as they are commonly used in clinical practice for the treatment of conditions such as attention deficit hyperactivity disorder (ADHD), dementia, and narcolepsy [193]. However, the broad category of cognitive enhancers includes many substances that, despite being legal, can have significant health consequences depending on patterns of use and abuse.

In 2013, the French Agency for Health and Food Safety issued an opinion on so-called energy drinks, which contain caffeine and other stimulants such as taurine. Their analysis of 257 cases of adverse reactions, including panic attacks, nervousness, and even epilepsy, demonstrated that high consumption of these drinks among high school students can be a marker for other risk behaviors, such as the intake of synthetic psychostimulants. Such behaviors can negatively impact adolescent development, health, and well-being, with effects comparable to those of illicit drugs [194]. Similarly, research conducted by Johns Hopkins University School of Medicine and the University of Vermont has explored the prevalence of caffeine addiction. This stimulant is often consumed even when it is medically inadvisable, such as during pregnancy or in individuals with cardiovascular disease or blood clotting disorders. Caffeine addiction has been identified as a condition known as Caffeine Use Disorder (CUD), in which excessive caffeine consumption leads to symptoms resembling stimulant drug overdose. Recognizing the growing body of evidence on this issue, the American Psychiatric Association has officially classified caffeine misuse disorder as a legitimate health concern, including it in Section III of the DSM-5 to encourage further research [195].

The increasing use of psychostimulants must therefore be examined within a broader sociocultural framework, considering prior psychological habits and the potential for relapses into addictive behaviors. Identifying the associated psychological and physiological symptoms would allow for a reassessment of the current regulations concerning their distribution, marketing, and use, promoting more stringent controls and greater safety measures. This issue is particularly urgent given that a significant portion of *smart drug* users are young students, including university students, who are otherwise healthy individuals turning to cognitive enhancers for reasons such as improving concentration, enhancing neurocognitive function, reducing stress, optimizing time, prolonging wakefulness, increasing free time, or simply out of curiosity [167].

The urgency of addressing this issue is reinforced by the increasing production and availability of these substances. According to the latest annual report from the EMCDDA—European Drug Report 2022: Trends and Developments, New Psychoactive Substances: Hazardous Substances Continue to Appear—as of the end of 2021, the agency was monitoring approximately 880 new psychoactive substances, with 52 first reported in Europe that year. Around 370 previously identified substances were detected on the market in 2020. National estimates of past year use of new psychoactive substances (excluding ketamine and gamma-hydroxybutyrate) among young adults (aged 15–34) ranged from 0.1% in Latvia to 5.1% in Romania. Among school-aged children, the 2019 ESPAD survey estimated the lifetime use of new psychoactive substances to be between 0.9% and 6.6%, with the lifetime use of synthetic cannabinoids ranging from 1.1% to 5.2% and synthetic cathinones from 0.2% to 2.5%. In 2020, 3-methylmethcathinone (3-MMC) was implicated in 38 cases of acute drug toxicity in five Euro-DEN Plus hospitals, while deaths involving synthetic cannabinoids were reported in Germany (9), Hungary (34), and Turkey (49). Low levels of 3-MMC were also detected in 10 European cities through drug-checking services. An analysis of 1166 used syringes collected by the ESCAPE network from seven European cities found synthetic cathinones in over half of all the syringes analyzed in Budapest and Paris [196].

Sociological studies have revealed the widespread and growing use of these substances, both occasionally and habitually. Today, *smart drugs* are commonly used not only in schools and universities, by both students and faculty, but also in other professional and social settings [197]. This phenomenon has fueled an ongoing bioethical debate regarding cognitive enhancement. The absence of strict regulations, combined with the widespread availability of smart drugs, has sparked a polarizing discussion among scientists. Some scholars advocate for enhancement, viewing cognitive enhancement as a social duty and a means of increasing control over mental and physical performance. From this perspective, smart drugs are perceived as a tool that fosters self-esteem and self-efficacy while responding to societal and cultural demands for greater productivity. In this framework, cognitive pharmacological enhancement is interpreted as a social phenomenon aligned with the ideals of power and success in a society that increasingly rewards intellectual performance and emotional control while marginalizing those who are deemed less productive [198].

Others, however, adopt a more moderate position, advocating for the responsible use of enhancement drugs while recognizing the need for regulation considering their increasing prevalence [199]. A critical issue frequently raised in support of this position concerns the long-term effects on mental and physical health. Many users exhibit symptoms like those of clinical conditions for which these substances are prescribed, while others, over time, experience increasing cognitive difficulties despite lacking a formal diagnosis of impairment [200].

Typically, smart drugs are obtained through prescriptions, over-the-counter purchases, online transactions, or from informal sources such as friends or family members. The clinical impact of ingesting cognitive enhancers can be significant, as these molecules influence multiple neurotransmitter systems in the brain, including the cholinergic, dopaminergic, noradrenergic, and serotonergic pathways [201]. As a result, while these substances may offer temporary cognitive benefits, their long-term impact on brain function, mental health, and overall well-being remains a subject of concern and warrants further research.

### 7.1. Cognitive Enhancers

The use of cognitive enhancement through psychiatric drugs represents the least invasive method to enhance or facilitate cognitive functions such as attention, concentration, and memory [202]. Certain drugs initially developed for the treatment of dementia and neurological disorders have been repurposed to improve intellectual performance.

One of the most used substances is methylphenidate. Research conducted by Volkow and colleagues [203] demonstrated that methylphenidate affects motivation, which can, in turn, influence academic performance by enhancing cognitive abilities and increasing students’ self-rated interest in monotonous tasks, such as mathematical exercises. A separate study highlighted that methylphenidate has one of the highest prescription rates among cognitive enhancers and is widely available through online sources offering the drug without a prescription to UK users [204]. University students are often drawn to methylphenidate for its perceived ability to enhance focus and attention [205]. The mechanism of action of methylphenidate involves the inhibition of catecholamine reuptake, leading to an increase in extracellular dopamine and norepinephrine levels. Originally prescribed for depressive states and psychosis associated with narcolepsy, it is now the most widely used treatment for ADHD, as it primarily improves working memory, memory consolidation, processing speed, and inhibitory control. It has also been shown to promote wakefulness in sleep-deprived individuals. However, its adverse effects include central nervous system disorders such as headaches, drowsiness, and tremors, as well as metabolic and nutritional imbalances, cardiovascular complications, psychiatric disturbances, skin reactions, anemia, and gastrointestinal issues [206].

Another class of widely used cognitive enhancers is amphetamines. These substances act as indirect agonists of the catecholaminergic system, primarily at the central level, by promoting the release of dopamine, norepinephrine, and serotonin. Their approved therapeutic uses include appetite regulation in obese patients and the treatment of mental and behavioral disorders such as narcolepsy and ADHD. Due to their remarkable ability to enhance both cognitive and physical performance, amphetamines rank among the primary pharmacological agents used as smart drugs for cognitive enhancement [207]. Studies have also shown that amphetamines improve episodic memory, working memory, and various aspects of attention in the general population. However, their use is associated with significant adverse effects, including tachycardia, irregular heartbeat, appetite suppression, hypertension, hallucinations, insomnia, and paranoid psychosis. Furthermore, their consumption significantly increases the risk of developing a full-blown addiction [203].

Modafinil is another frequently used cognitive enhancer, though its mechanism of action is not yet fully understood. It is believed to inhibit catecholamine reuptake while increasing extracellular dopamine levels in the front striatal region and norepinephrine levels in the frontal regions. Additionally, it may influence several other neurotransmitters, including serotonin, glutamate, and GABA. Modafinil is widely praised for its ability to enhance reaction time, logical reasoning, and problem-solving skills. It is primarily prescribed for the treatment of narcolepsy, as it effectively increases alertness and wakefulness. However, its use is associated with side effects such as dizziness, drowsiness, asthenia, insomnia, blurred vision, mood irritability, loss of appetite, nausea, and xerostomia [208].

The category of nootropics—from the Greek noos (mind) and tropein (to supervise)—includes substances commonly referred to as smart drugs or lifestyle drugs. These compounds work by either directly or indirectly enhancing neurochemical release, thereby improving learning processes or stimulating nerve growth. One of the most well-known nootropic families is the racetams, which includes compounds such as piracetam and oxiracetam [209]. Piracetam belongs to a group of nootropic drugs known for their ability to enhance brain cell metabolism and energy production [210]. Although piracetam is officially classified as a nootropic, its cognitive-enhancing effects in healthy individuals are considered moderate. However, racetam molecules are being explored for potential therapeutic applications in neurodegenerative conditions such as Alzheimer’s disease, narcolepsy, ADHD, Parkinson’s disease, and age-related cognitive decline [211].

Among the drugs used to treat Parkinson’s disease, levodopa and tolcapone have also been investigated for their cognitive-enhancing properties. Levodopa is a biosynthetic precursor of dopamine, norepinephrine, and epinephrine and has been found to improve certain cognitive functions, including coding skills. However, its use is associated with several contraindications, including “wearing-off” effects, on–off periods, nausea, vomiting, appetite loss, anorexia, orthostatic hypotension, arrhythmias, psychiatric disturbances, and dyskinesias. Tolcapone, a selective inhibitor of catechol-O-methyltransferase (COMT) in the CNS, has been found to significantly enhance executive functions and episodic verbal memory. However, its use is associated with multiple side effects, including dystonia, headaches, dizziness, sleep disorders, excessive dream activity, drowsiness, mental confusion, hallucinations, appetite loss, nausea, and dyskinesia. Despite its cognitive benefits, the potential risks associated with tolcapone highlight the challenges in repurposing neurological drugs for cognitive enhancement [212].

### 7.2. Mood Enhancers

Smart drugs also include medications commonly used to treat depression, which can be employed to alter mood, emotional experiences, and personality states, reducing feelings of sadness and promoting a sense of happiness. But why is mood considered within the broader discussion of cognitive enhancement? This is because cognitive functions such as attention, memory, and learning are significantly influenced by emotional states. Thus, cognitive enhancement is often discussed in conjunction with emotional enhancement (mood enhancement), as the two are dynamically interconnected and mutually influential. Among the active ingredients that fall into this category, gabapentin and pregabalin stand out [207].

The mechanism of action of gabapentin involves its interaction with voltage-gated calcium channels, leading to a reduction in intracellular calcium ion concentration. This, in turn, decreases the activity of excitatory neurotransmitters such as glutamate, norepinephrine, and substance P while amplifying the activity of the inhibitory neurotransmitter GABA. Gabapentin is primarily used for the treatment of partial seizures with or without secondary generalization, neuropathic pain, bipolar disorder, anxiety disorders, and as a mood stabilizer. Additionally, it is sometimes used as an alternative to opioid substances, as it appears to produce lower levels of tolerance and addiction. However, potential adverse effects include mental confusion, drowsiness, and dizziness. In some cases, an increase in the frequency of epileptic seizures or the onset of new types of seizures, as well as suicidal ideation and behavior, has been reported [206].

Similarly, pregabalin is often used as an alternative to opioids, both due to its lower cost and because it is believed to cause less tolerance and addiction. It is commonly combined with opioid substances to achieve a synergistic effect. While pregabalin is primarily prescribed for the treatment of central and peripheral neuropathic pain, it is also used as an anxiolytic and hypnoinducer. Like gabapentin, its mechanism of action involves reducing the intracellular calcium ion concentration, which decreases the activity of neurotransmitters such as glutamate, norepinephrine, and substance P. However, its use may induce adverse effects, including lethargy, muscle spasms, and disturbed peripheral vision [207].

Finally, certain substances typically used for blood pressure and heart regulation can also be applied to manage anxiety; enhance psychological well-being; and produce feelings of euphoria, relaxation, and perceptual amplification. Propranolol, a beta-blocker, acts as a competitive antagonist at receptor sites for endogenous catecholamines such as adrenaline and norepinephrine on beta-adrenergic receptors. It is primarily indicated for the treatment of arterial hypertension, angina pectoris, tachycardia, and myocardial infarction, but it also has psychiatric applications, including the treatment of post-traumatic stress disorder (PTSD) and essential tremor. Propranolol is commonly used as a cognitive enhancer due to its anxiolytic effects, helping to reduce fear and anxiety. However, it can also produce adverse effects, including sleep disturbances, insomnia, nightmares, hyperthyroidism, Raynaud’s syndrome, peripheral vascular disorders, and an increased risk of diabetes mellitus [207].

### 7.3. Neuronal and Ethical Costs

Cognitive enhancement is currently one of the most debated topics in neuroscience. The aim of this study is to outline the potential risks associated with the use and abuse of major cognitive-enhancing substances, particularly among healthy individuals, young people, and adolescents. While smart drugs may offer temporary cognitive improvements, they likely come with both neuronal and ethical costs.

Dysregulation of the glutamate system can impair behavioral flexibility, potentially leading to the development or reinforcement of addictive behaviors. Alterations in glutamate concentrations, which play a crucial role in excitatory signaling between neurons, can disrupt synaptic function. Glutamate is essential for memory formation, attention regulation, and emotional control. Additionally, it plays a critical role in neuroplasticity, learning, and motor control. Through this amino acid, neuronal differentiation, migration, and the formation of new synaptic connections occur, contributing to overall brain health [213].

Dopamine and norepinephrine, the primary neurotransmitters involved in cognitive enhancement, do not exhibit linear effects on cognition. Instead, their impact follows an inverted U-curve, meaning that both excessive and insufficient levels can be detrimental. This implies that healthy individuals using cognitive enhancers risk exceeding the optimal neurotransmitter levels, leading to hyperdopaminergic and hyper noradrenergic states. Consequently, instead of enhancing the performance, they may experience a decline in cognitive function, impairing precisely the abilities they sought to improve [174]. Certain brain areas, such as the frontal lobes and higher executive function regions, complete their development in the later stages of adolescence. These areas are responsible for advanced cognitive tasks, including planning, goal setting, problem-solving, and self-regulation [212]. The widespread use of smart drugs among students and university attendees may interfere with the proper maturation of these cognitive abilities, potentially impairing their long-term development.

Recent studies have highlighted the potential detrimental effects of stimulant exposure on young, healthy individuals, emphasizing that performance enhancement may come at a cost to the developing brain. One of the key mechanisms ensuring efficient brain function is synaptic pruning. This process strengthens highly active synapses while eliminating weaker ones through axon retraction, following the principle of “use it or lose it”. The goal of synaptic pruning is to refine neuronal networks, enhancing the brain’s efficiency. During adolescence, the number of synaptic connections naturally decreases due to pruning, which is influenced by environmental factors and is widely considered a fundamental aspect of learning. At first glance, increased neuroplasticity might seem advantageous, as it can lead to faster learning and improved cognitive abilities. However, excessive plasticity can result in heightened synaptic activity across the brain, reducing the overall efficiency. Impaired synaptic pruning has been associated with neurodevelopmental disorders such as autism spectrum disorder. Excessive connectivity can increase overall brain activation while reducing the selectivity of neural responses, thereby significantly decreasing the signal-to-noise ratio. Unregulated brain plasticity could therefore pose a significant risk, and substances that enhance synaptic strength by intensifying plasticity and promoting dendritic spine growth may contribute to conditions resembling autism.

Although no studies have yet confirmed this phenomenon in humans, the uncontrolled administration or widespread availability of these substances increases the risk of their misuse. Exceeding safe dosages could potentially lead to neuronal damage, highlighting the need for greater regulation and awareness regarding the risks of cognitive enhancers [174].

## 8. Conclusions

Smart drugs are a growing public safety concern, not only due to their often complex and variable composition but also because they are frequently consumed in combination with other substances, increasing the risk of dangerous drug interactions. Additionally, their side effects remain only partially understood, making them a significant health risk, particularly given their rising popularity among young people and working professionals.

Currently, there are few longitudinal and experimental studies examining the nature and effects of smart drugs, partly due to ethical constraints. However, given their increasing use, it is crucial to adopt a multidisciplinary approach to properly classify new herbal blends, integrating these data into institutional web repositories, such as those managed by the EUDA. Furthermore, conducting new studies on animal models is essential to investigate the real effects of these substances, their potential for addiction, their impact on neuronal circuits, and the associated psychological implications. Such research would facilitate a faster and more accurate risk assessment of herbal products and smart drugs in general, ultimately contributing to public health protection. To support future research and monitoring efforts, Table 4 summarizes proposed experimental activities aimed at improving the detection, characterization, and risk assessment of new psychoactive substances.

Considering the risk factors and motivations that drive college students and young adults to use cognitive enhancers, it is essential to raise awareness about the harm associated with their use and abuse. Efforts should focus on debunking myths about the so-called “safe” use of these substances and addressing cognitive enhancement at an early stage through education and the implementation of appropriate cognitive strategies. Taking such preventive public health measures is critical in mitigating the potential risks associated with smart drug consumption.

Furthermore, given the increasing prevalence and diversity of smart drugs available on the market, it is imperative to underline the importance of continued research into their long-term effects on brain function and psychological health. Although preliminary evidence from animal studies strongly suggests significant long-term neuroplastic and neurotoxic effects associated with repeated exposure to substances such as amphetamines, synthetic cathinones, and cannabinoids, our understanding remains incomplete. There is an urgent need to pursue extensive longitudinal studies employing advanced neuroimaging technologies, molecular biology techniques, and robust cognitive and behavioral assessments to elucidate the comprehensive consequences of chronic smart drug consumption on neural circuits, cognition, and mental health.

In addition, future research should prioritize the identification of biomarkers indicative of vulnerability to cognitive impairment and psychological disorders stemming from the prolonged use of these substances. Identifying individual susceptibility and resilience factors may aid in developing personalized prevention strategies, therapeutic interventions, and public health policies aimed at reducing the detrimental impacts of smart drug use.

Ultimately, addressing these knowledge gaps through multidisciplinary research efforts will be crucial in guiding informed decision-making among both consumers and policymakers and in safeguarding public health from the evolving threats posed by the continuous emergence of novel psychoactive substances.

## Figures and Tables

**Table 1:** Overview of the available literature on analytical methods used for the determination of plant-derived smart drugs as single molecules or class.

Molecule	LLE	SPE (Cartridge)	SPE (Elution Solvent)	Analytical Method	Stationary Phase	Mobile Phase (Solvent/Gas Carrier)	MS (*m*/*z*)	UV λ_max_ (nm)	IR λ_max_ (vsm^−1^)	Ref.
Caffeine	Chloroform + dichloromethane	MIP	Acetonitrile + acetic acid	HPLC–DAD	C18	Water + acetic acid + acetonitrile	194	272	1700	[128,129]
Polyphenols	Ethyl acetate + methanol	XTR Chromabond	Ethyl acetate	HPLC–UV HPLC–DAD	C18	Acidified water + methanol or acetonitrile		320–380 (band I) 240–270 (band II)	3500–3200	[130]
Huprazine A	Ethyl ether + chloroform	MIP1/PIN1	Methanol + ammonia	HPLC–DAD	C18	Ethanol 95% Ethanol 30% Ethanol 40%	243	305	3368–3267	[131,132]
	Ethanol + isopropanol			LC–MS/MS	C18	Methanol + water + ammonium formate	243			[133]
Withaferin A	Methyl tert-butyl ether			LC–MS/MS	C18	Methanol + ammonium acetate	471	214	1692	[134]
Schisandrin	Water			LC–MS/MS	C18	Water + formic acid + acetonitrile	432			[135]
	Methyl Ether + tertbutyl ether + dichloromethane + ethyl acetate			UFLC–MS/MS	Shim-pack XR-ODS	Water + formic acid + acetonitrile				[136]
		HLB	Methanol + acetic acid + ammonia	HPLC–DAD	C18 Bondclone	Methanol + water				[137]
Syringin	Methanol			HPLC–DAD	C18	Water + phosphoric acid + acetonitrile	431 209 432 371 433	200–370		[138]
		C18	Water + methanol	HPLC–DAD	Symmetry C18	Methanol + water				[139]
Geraniol	Pentane + diethyl ether			GC–MS	ZB-Wax	Helium	77 80 93 121 143		2900	[140]
Monoterpenols	Dichloromethane			GC–MS	DB-FFAP	Helium				[141]
Arecoline	Chloroform/isopropane			HPLC–MS	C18	Ammonium acetate + acetonitrile	156 140 118			[142]
Dehydrocorydaline	Water-saturated n-butanol ethyl acetate			HPLC–ESI–QTRAP–MS	Silica column	Helium	351			[143]
Lysergamide	Methanol			GC–MS	DB-1	Helium	267			[144]
				UHPLC–QTOF-MS/MS	C18	Acetonitrile + formic acid				
				HPLC–DAD	Synergi Fusion–RP	Acetonitrile				
				LC–MS	PPFP	Water + formic acid Acetonitrile + formic acid				
Ephedrine		MIP	Acetonitrile	HPLC	Stainless steel	Acetonitrile + sodium acetate + methanol	148 117 133			[145,146]
	Toluene-benzene			HPLC	C18	Acetonitrile	115 70			
α-asarone βasarone	*n*-hexane			HPLC	C18	Water + methanol	208	254		[147]
				HS–GC/MS	Fused silica	Helium				[148]
Atropine alkaloids	*n*-hexane, Diethyl ether			GC–MS GC–FID	RTX-5 RTX-5	Helium Argon				[149]
		MIP	Acetonitrile + acetic acid	HPLC	C18	Acetonitrile Aqueous ammonium acetate				[150]
Isoquinoline alkaloids		GP-resin	Methanol Chloroform	HPLC	C18	Trifluoroacetic acid + acetonitrile	129 51 102 50 76	280		[151]
	Ammonium hydroxide Dichloromethane			HPLC–DAD–MS/MS	C18	Trifluoroacetic acid + methanol				[152]
Indole alkaloids	Chloroform, methanol			HPLC	C8, C18 and phenyl–hexyl	Monosodium phosphate + citric acid	117 89 118 63 116			[153]
		MIP	Methanol Acetonitrile	HPLC–UV	XTerra MS C18	Acetonitrile + acetic acid + ammonia buffer				[154]

**Table 2:** Overview of the available literature on analytical methods used for the determination of synthetic smart drugs (cannabinoids, cathinones, and designer drugs).

Molecule	LLE	SPE (Cartridge)	SPE (Elution Solvent)	Analytical Method	Stationary Phase	Mobile Phase (Solvent/Gas Carrier)	MS (*m*/*z*)	UV λ_max_ (nm)	Ref.
Classical cannabinoids	Methanol/chloroform			HPLC–DAD	C18	Methanol + water			[155]
	Ethanol			HPLC–DAD	C18	Acetonitrile + formic acid			[156]
				HPLC–DAD	RX-C18	Water + acetonitrile			
						Acetonitrile + trifluoroacetic acid			
				HPLC–DAD	EC-C18	Acetonitrile + formic acid			
		Strata phenyl	Acetonitrile	LC−ESI−MS/MS	Kinetex core-shell biphenyl	Ammonium formate + acetonitrile			[157]
Hybrid cannabinoids	Ether			HPLC–UV	Chiracel OD	Propanol + hexane		254	[158]
				IR–HPLC	Silica gel	Ethyl Acetate + hexane			
Aminoalkylindoles	n-hexane/ethyl acetate			LC–MS/MS	Phenyl hexyl	Formic acid + ammonium formate + methanol			[159]
Synthetic cathinones		MIP	Methanol	LC–MS/MS	Kinetex C18	Acetonitrile + formic acid			[160]
Mephedrone	Hexane			GC–MS	Phenylmethylsiloxane	Helium	130 133		[161]
	Hexane			LC–MS/MS	C18	Acetonitrile			[162]
Methcathinones	Water			LC–MS/MS	C18	Acetonitrile + formic acid	58	210	[163]
DMAA	Dichloromethane			GC–MS	HP-5	Helium	44		[164]
MXE	Acetonitrile–ethanol			LC–MS/MS	C18	Formic acid + ammonium + methanol	190		[165]
		Clean Screen	Methylene chloride + isopropanol + ammonium hydroxide	GC–MS	ZB-50	Helium			[166]

**Table 3:** Summary of major psychological effects and adverse effects of different psychoactive drugs and drug classes.

Drug/Drug Class	Major Psychological Effects	Major Adverse Effects	References
Amphetamines (Methamphetamine and Amphetamine-based ligands)	Euphoria, increased alertness, heightened cognitive performance, increased self-confidence, improved mood, and increased sociability	Neurotoxicity, degeneration of dopaminergic neurons, cognitive impairment, psychosis resembling schizophrenia symptoms, addiction potential, and long-term neuroplastic changes, including reduced NGF and BDNF levels	[180,181,182]
Cocaine	Intense euphoria, increased energy levels, elevated mood, enhanced confidence, reduced feelings of fatigue, and heightened sense of reward	Neurotoxicity, significant addiction risk, cognitive deficits, changes in glutamatergic neurotransmission, dopaminergic neurodegeneration, and impaired synaptic plasticity involving AMPA receptors	[182,183]
Methylphenidate (MPH)	Enhanced attention, improved cognitive functions, increased wakefulness and alertness, improved working memory, and reduced impulsivity	Dopaminergic neuron loss, increased oxidative stress, neuroinflammation, altered neuroplasticity, heightened sensitivity to neurotoxic effects, and potential long-term neurodegenerative changes	[183,184,185]
Synthetic Cannabinoids	Relaxation, euphoria, altered sensory perception, sedation, and feelings of dissociation	Severe anxiety, paranoia, psychosis, hallucinations, tachycardia, neurotoxicity, cognitive impairments, risk of addiction, and cardiovascular problems	[69,186,187]
Synthetic Cathinones (“Bath Salts”)	Increased sociability, euphoria, stimulation, heightened energy, and increased libido	Agitation, severe anxiety, psychosis, paranoia, aggression, hallucinations, cardiovascular toxicity, neurotoxicity, and risk of addiction	[5,6,85]
DMT-based Substances (Ayahuasca and synthetic DMT analogs)	Altered states of consciousness, spiritual or mystical experiences, visual and auditory hallucinations, and altered perception of time	Anxiety, paranoia, panic reactions, nausea, vomiting, increased heart rate and blood pressure, and risk of serotonin syndrome	[188,189,190]

**Table 4:** Proposed future experimental activities for investigating new psychoactive substances (NPS).

Objective	Experimental Activity	Methodology	Expected Outcome
Identification of emerging NPS	Molecular identification (DNA barcoding and metabarcoding)	DNA extraction, PCR amplification, sequencing, and bioinformatics analysis	Accurate identification of plant and synthetic origin substances
Morphological characterization of NPS	Micro-morphological analyses (light microscopy, SEM, and TEM)	Microscopic examination of samples at various magnifications and resolutions	Detailed morphological profiles to distinguish substances
Chemical profiling and characterization	Chemical analyses (GC-MS, HPLC, and LC-MS/MS)	Extraction and quantification of bioactive compounds	Comprehensive chemical fingerprints of identified substances
Toxicological risk assessment	In vitro and in vivo toxicological studies	Cytotoxicity assays and animal model evaluations	Risk profiles and safety data on NPS
Development of a comprehensive database	Integration of molecular, morphological, and chemical data into an accessible database	Creation of a centralized digital platform for data storage and retrieval	Enhanced monitoring, rapid identification, and dissemination of information about new psychoactive substances

## Data Availability

No new data were created or analyzed in this study. Data sharing is not applicable to this article.

## Abbreviations

The following abbreviations are used in this manuscript:

EMCDDA	European Monitoring Centre for Drugs and Drug Addiction
EUDA	European Union Drugs Agency
ISS	Istituto Superiore di Sanità
SNAP	System Network on New Psychoactive Substances
AIFA	Italian Medicines Agency
LSD	Lysergic Acid Diethylamide
LSA	Lysergic Acid Amide
CNS	Central Nervous System
DMT	N,N-Dimethyltryptamine
MAO	Monoamine Oxidase
MAOIs	Monoamine Oxidase Inhibitors
5-OH-DMT	5-Hydroxy-N,N-Dimethyltryptamine
5-MeO-DMT	5-Methoxy-N,N-Dimethyltryptamine
Δ^9^-THC	Δ-9-Tetrahydrocannabinol
SCs	Synthetic Cannabinoids
CB1	Cannabinoid Receptor 1
CB2	Cannabinoid Receptor 2
HU-210	(6aR,10aR)-9-(Hydroxymethyl)-6,6-Dimethyl-3-(2-Methyloctan-2-yl)-6a,7,10,10a-Tetrahydrobenzo[c]chromen-1-ol
CP-47947	5-(1,1-Dimethylheptyl)-2-[(1R,3S)-3-Hydroxycyclohexyl]-Phenol
AM-4030	(6S,6aR,9R,10aR)-9-(Hydroxymethyl)-6-[(E)-3-Hydroxyprop-1-Enyl]-6-Methyl-3-(2-Methyloctan-2-yl)-6a,7,8,9,10,10a-Hexahydrobenzo[c]chromen-1-ol
AM-1220	(Naphthalen-1-yl)[1-[(1-Methylpiperidin-2-yl)Methyl]-1H-Indol-3-yl]Methanone
THJ-018	Naphthalen-1-yl[1-(Pent-1-yl)-1H-Indazol-3-yl]Methanone
THJ-2201	1-(5-Fluoropent-1-yl)-1H-Indazol-3-ylMethanone
5F-APINACA	N-(1-Adamantyl)-5-Fluoropentyl-1H-Indazole-3-Carboxamide
MDMB-FUBINACA	Methyl 2-[1-(4-Fluorobenzyl)-1H-Indazole-3-Carboxamido]-3,3-Dimethylbutanoate
ADB-FUBINACA	N-(1-Amino-3,3-Dimethyl-1-Oxobutan-2-yl)-1-(4-Fluorobenzyl)-1H-Indazole-3-Carboxamide
AB-CHMINACA	N-(1-Amino-3-Methyl-1-Oxobutan-2-yl)-1-(Cyclohexylmethyl)-1H-Indazole-3-Carboxamide
MAB-CHMINACA	N-(1-Amino-3,3-Dimethyl-1-Oxobutan-2-yl)-1-(Cyclohexylmethyl)-1H-Indazole-3-Carboxamide
AM-356	N-(2-Hydroxy-1R-Methylethyl)-5Z,8Z,11Z,14Z-Eicosatetraenamide
EPH	Ephedrone
MDPV	3,4-Methylenedioxypyrovalerone
βk-MDMA	3,4-Methylenedioxy-N-Methylcathinone
MDPBP	Methylenedioxy-α-Pyrrolidinobutyrophenone
4-MMC	4-Methylmethcathinone, 4-Methylephedrone
βk-MBDB	1,3-Benzodioxol-5-yl)-2-(Methylamino)Butan-1-one
PMMA	Paramethoxymethamphetamine
2C-B	4-Bromo-2,5-Dimethoxyphenethylamine
2C-I	4-Iodo-2,5-Dimethoxyphenethylamine
2C-T-7	2,5-Dimethoxy-4-(n)-Propylthiophenethylamine
DOB	2,5-Dimethoxy-4-Bromoamphetamine
DOI	2,5-Dimethoxy-4-Iodoamphetamine
25I-NBMOe	2-(4-Iodo-2,5-Dimethoxyphenyl)-N-[(2-Methoxyphenyl)Methyl]Ethanamine
4-APB	4-(2-Aminopropyl) Benzofuran
5-APB	5-(2-Aminopropyl) Benzofuran
6-APB	6-(2-Aminopropyl) Benzofuran
DiTP	Di-Isopropyltryptamine
4-OH-DiPT	4-Hydroxy-N,N-Diisopropyltryptamine
BZP	1-Benzylpiperazine
mCPP	1-(m-Chlorophenyl) Piperazine
TFMPP	1-(m-Trifluoromethylphenyl) Piperazine
2-DPMP	2-Diphenylmethylpiperidine
D2PM	Diphenyl-2-Pyrrolidinyl-Methanol
MOTUs	Molecular Operational Taxonomic Units
BLAST	Basic Local Alignment Search Tool
BOLD-IDS	Barcode of Life Database Identification System
SEM	Scanning Electron Microscopy
TEM	Transmission Electron Microscopy
EDS	X-ray Energy Dispersive System
LOD	Limit of Detection
LOQ	Limit of Quantification
SPE	Solid-Phase Extraction
RP-HPLC-DAD	Reverse-Phase High-Performance Liquid Chromatography Coupled with Diode Array Detector
LC-MS	Liquid Chromatography Coupled with Mass Spectrometry
LC-MS/MS	Liquid Chromatography Tandem Mass Spectrometry
GC-MS	Gas Chromatography Coupled with Mass Spectrometry
GC-FID	Gas Chromatography Coupled with Flame Ionization Detector
ADHD	Attention Deficit-Hyperactivity Disorder
CUD	Caffeine Use Disorder
3-MMC	3-Methylmethcathinone
CE	Cognitive Enhancers
COMT	Catechol-O-Methyltransferase
PTSD	Post-Traumatic Stress Disorder

## Drug Compounds Index

25B-NBOMe	Section 3.4
25C-NBOMe	Section 3.4
25I-NBOMe	Section 3.4
2C-B	Section 3.4
2C-B	Section 3.4
2C-I	Section 3.4
2C-I	Section 3.4
2C-T-7	Section 3.4
2C-T-7	Section 4
2-DPMP	Section 3.4
3-MMC	Section 7
4-APB	Section 3.4
4-MMC	Section 3.2
4-OH-DiPT	Section 3.4
5-APB	Section 3.4
5-MeO-DMT	Section 3.1.2
5-MeO-DMT	Section 3.1.2
5-OH-DMT	Section 3.1.2
6-APB	Section 3.4
AB-CHMINACA	Section 3.2
ADB-FUBINACA	Section 3.2
AM-356	Section 3.2
AM-4030	Section 3.2
Atropine	Section Tropane Alkaloids
Bufotenin	Section 3.1.2
BZP	Section 3.4
Cathinone	Section 1, Section 3.3, Section 6, Section 7 and Section 8
Cocaine	Section 1, Section 3.2, Section 3.4 and Section 7; Section Indole Alkaloids
CP-47947	Section 3.2
D2PM	Section 3.4
D2PM	Section 3.4
DiTP	Section 3.4
DMT	Section 3.1.2 and Section 6
DOB	Section 3.4
DOI	Section 3.4
Ephedrine	Section Pseudoalkaloids; Section 5
Ephedrone	Section 3.2
HU-210	Section 3.2
Hyoscyamine	Section Tropane Alkaloids
K2	Section 1 and Section 3.2
Kratom	Section 1; Section Indole Alkaloids
LSA	Section Ergoline Alkaloids
LSD	Section Ergoline Alkaloids; Section 3.1.2
mCPP	Section 3.4
MDMA	Section 1, Section 3.2 and Section 3.4
MDPV	Section 3.3
Mephedrone	Section 3.3
Methamphetamine	Section 3.3; Section 3.4
Methcathinone	Section 3.3
NBOMe	Section 3.4
PMMA	Section 3.4
Scopolamine	Section Tropane Alkaloids
Spice	Section 1, Section 3.2 and Section 4.2
TFMPP	Section 3.4
THC	Section 3.2
THJ-018	Section 3.2
THJ-2201	Section 3.2
βk-MBDB	Section 3.3
βk-MDMA	Section 3.2
